# Skin swabbing is a refined technique to collect DNA from model fish species

**DOI:** 10.1038/s41598-020-75304-1

**Published:** 2020-10-23

**Authors:** Ceinwen A. Tilley, Hector Carreño Gutierrez, Marion Sebire, Oluwapelumi Obasaju, Florian Reichmann, Ioanna Katsiadaki, Iain Barber, William H. J. Norton

**Affiliations:** 1 Department of Neuroscience, Psychology and Behaviour, College of Life Sciences, University of Leicester, Leicester, LE1 7RH UK; 2 Centre for Environment, Fisheries and Aquaculture Science, Weymouth Laboratory, Barrack Road, The Nothe, Weymouth, Dorset DT4 8UB UK; 3 Division of Pharmacology, Otto Loewi Research Centre for Vascular Biology, Immunology and Inflammation, Medical University of Graz, Graz, Austria; 4 School of Animal, Rural and Environmental Sciences, Nottingham Trent University, Brackenhurst Campus, Brackenhurst Lane, Southwell, NG25 0QF UK; 5 Institute of Biology, Department of Genetics, ELTE Eötvös Loránd University, Budapest, Hungary

**Keywords:** Zoology, Animal behaviour, Animal physiology

## Abstract

Model fish species such as sticklebacks and zebrafish are frequently used in studies that require DNA to be collected from live animals. This is typically achieved by fin clipping, a procedure that is simple and reliable to perform but that can harm fish. An alternative procedure to sample DNA involves swabbing the skin to collect mucus and epithelial cells. Although swabbing appears to be less invasive than fin clipping, it still requires fish to be netted, held in air and handled—procedures that can cause stress. In this study we combine behavioural and physiological analyses to investigate changes in gene expression, behaviour and welfare after fin clipping and swabbing. Swabbing led to a smaller change in cortisol release and behaviour on the first day of analysis compared to fin clipping. It also led to less variability in data suggesting that fewer animals need to be measured after using this technique. However, swabbing triggered some longer term changes in zebrafish behaviour suggesting a delayed response to sample collection. Skin swabbing does not require the use of anaesthetics and triggers fewer changes in behaviour and physiology than fin clipping. It is therefore a more refined technique for DNA collection with the potential to improve fish health and welfare.

## Introduction

Changes to the health and welfare of animals can alter their behaviour and physiology^1^. Animal welfare is also an ethical responsibility for researchers^2,3^. It is therefore imperative that healthy animals are used in research to collect valid, reliable and reproducible results^4–6^.

Fish are considered useful alternatives to mammals within the principles of replacement, reduction and refinement (‘3Rs’) for the use of animals in research. Historically, research into fish welfare has not received much attention and has lagged behind studies in mammals^1,7,8^ although the situation has improved in recent years^9–11^. Fish are also subject to the 3Rs principles. There is increasing awareness that fish may experience pain, stress or lasting harm as a consequence of invasive procedures^11^. This has driven the development of alternative, replacement methodologies—such as the use of cell or tissue cultures—and the refinement of existing techniques, for example by applying anaesthesia and analgesia^11,12^.

Many laboratory studies use small-bodied model fish species such as the zebrafish *Danio rerio* and the three-spined stickleback *Gasterosteus aculeatus*. Advantages of these species include their small size, ease of maintenance in the laboratory and similarities to other vertebrates^9,12–15^. DNA is frequently collected from these animals to facilitate identification by genotyping, and this is usually achieved by fin clipping under non-terminal anaesthesia^16^. The number of fish used in research is rising steadily. In the United Kingdom, fish accounted for 17% of regulated procedures in 2018^17^. A recent survey indicated that 85% of zebrafish labs use fin clipping to collect DNA, a licenced procedure under the UK Animals (Scientific Procedures) Act 1986^18^.

Despite its popularity, the effect of fin clipping on fish welfare has only recently been investigated^3,19^. Tissue biopsy may negatively affect fish leading to infection and altering survival, growth and behaviour^20^. Fish that have undergone fin clipping show increased anxiety-like behaviour, including increased ventilation^21^, reduced activity^6,21,22^, increased time at the bottom of a tank^3,6,19,21^ and decreased feeding^3^. Fin clipping can also induce release of the primary stress hormone cortisol^19^. Fish may be negatively affected by any or all of the constituent parts of the fin clipping procedure, which comprises capture in a net, non-lethal anaesthesia, exposure to air, removal of a small section of caudal fin with a scalpel blade and subsequent recovery. For example, the initial stress of netting a fish and exposing it to air can cause elevated cortisol levels^23,24^, and zebrafish also actively avoid exposure to anaesthetics such as MS-222 and benzocaine^25^ which can trigger stress responses^26^. Since such changes can dramatically affect the quality of experimental data^5^, efforts to improve and refine techniques for DNA collection have increased^27,28^. Developing an alternative to fin clipping will not only benefit the health and welfare of fish but may also improve the reliability, and therefore the repeatability, of scientific data collected^29^ with the potential to reduce the number of animals used.

Alternative methods of DNA collection such as scale, barbel, muscle, blood, mucus and sperm sampling have been gaining popularity^30^. Some of these techniques are less invasive than fin clipping, with the potential to improve welfare and remove the need for legal regulation. For example, skin swabbing can be performed without using anaesthesia, whereas tissue biopsy requires anaesthesia according to UK regulations^27^.

Skin swabbing removes the mucus layer from a fish’s skin, and the DNA collected by this technique likely comes from epithelial cells that have been sloughed off. Fish mucus is produced by goblet cells. It is a viscous colloid substance that contains water, antibacterial enzymes and high molecular weight thread-like glycoproteins called mucins^31^. Mucus has rheological and viscoelastic properties^31^ and is important for disease resistance, respiration, ionic and osmotic regulation, reproduction, excretion, communication, feeding and nest building depending upon the species^32^. Mucus also provides a physical and chemical barrier against pathogens. It contains a number of innate immunity factors such as lysozyme, immunoglobulin, lectins and C-reactive protein, and prevents both the adherence of pathogens and invasion of parasites^31^. Skin swabbing has been used successfully in many fish species including African cichlids *Neolamprologus pulcher*^27^, Atlantic cod *Gadus morhua*^33^ bluegill sunfish *Lepomis macrochirus*^30^, Nile tilapia, *Oreochromis niloticus*^20^, three-spined sticklebacks^34^ and zebrafish^28^. Intuitively, swabbing appears to be less invasive than fin clipping since it does not require the use of anaesthetic or the need to remove tissue. Although the technique has been validated with regards to DNA yield, PCR amplification and the potential for cross-contamination^28,35^, the actual welfare benefit of swabbing over fin clipping remains untested. A direct comparison of the impact of both techniques on fish welfare indicators is essential to validate swabbing as a refinement and aid its adoption by researchers.

In this study we use behaviour, stress axis response and other condition indicators to investigate the hypothesis that swabbing is less harmful to fish than fin clipping. We examine the different steps used in each method to highlight factors that may alter health or welfare. Our data suggest that swabbing is less invasive and stressful to fish than fin clipping, confirming that swabbing is a refined method to collect DNA from fish.

## Methods

### Fish stocks and husbandry

Zebrafish and sticklebacks were kept in accordance with Institutional guidelines for animal welfare. All work was conducted under a UK Home Office licence and was approved by a local Animal Welfare and Ethical Review Body (AWERB) committee at the University of Leicester.

#### Three-spined sticklebacks

Stocks of F2 generation lab bred three-spined sticklebacks (*Gasterosteus aculeatus*) were generated by in vitro fertilisation in July 2017, as described by Barber and Arnott^36^. The parental background was a wild freshwater population originally collected from the River Welland (Market Harborough, Leicestershire, UK) in 2015. The sticklebacks used in this study had an average length of 36.74 ± 2.98 mm and an average weight of 0.67 ± 0.2 g. Adult sticklebacks were fed an ad libitum diet of defrosted bloodworm (*Chironomus* sp. larvae) until the start of experiments. Groups of fish were pooled into large stock tanks (27 L) in a dedicated fish facility at the University of Leicester. The tanks housed 40 fish on a circulating system (Xenoplus systems, Techniplast) with a flow rate of 2 tank changes per hour. The system water was made from reverse osmosis water with marine salts added (Instant Ocean). The water parameters were pH ~ 7.1, 0 ppm ammonia, 0 ppm nitrate, ~ 4 ppm nitrite and ~ 4000 DKH conductivity. Temperature and light–dark conditions were adjusted periodically to simulate natural seasonal variation until March 2018. Fish were then kept at 12 ± 1 °C on a 12:12 h light:dark cycle (i.e. March conditions) to maintain non-breeding conditions for the duration of the experiments, which were conducted from May 2018 onwards. Therefore, all experimental studies were carried out using non-reproductive individuals. Males and females were visually identical, thus avoiding confounding factors associated with territorial and courtship behaviours.

#### Zebrafish

AB wild type zebrafish (*Danio rerio*) were generated by in-crossing parental stock maintained at the University of Leicester. The zebrafish used in this study had an average length of 34.99 ± 1.66 mm and an average weight of 0.38 g ± 0.08 g. The stock tanks housed 40 fish in 8 L tanks on a circulating system (ZebTEC multi-link water treatment unit, Techniplast) with a flow rate of 7.6 L per tank per hour (equating to around one tank change per hour). The system water was made from reverse osmosis water with Instant Ocean salts (Aquarium Systems, UK) added. The water parameters were pH ~ 7.1, 0 ppm ammonia, 0 ppm nitrate, ~ 4 ppm nitrite and ~ 525 DKH conductivity. The fish were maintained at 28 ± 1 °C and in a 14:10 h light:dark cycle.

### Experimental design

The experimental design and fish numbers for each group were determined by the NC3Rs Experimental Design Assistant EDA (https://eda.nc3rs.org.uk/) (see Supplemental Material)^37^ and power analysis. The total number of individuals used was 567 sticklebacks (27 × 7 groups × 3 independent replicates) and 630 zebrafish (30 × 7 groups × 3 independent replicates). One week prior to experimentation fish were caught and randomly distributed into seven tanks (27 sticklebacks in 13.4 L tanks and 30 zebrafish in 3.5 L tanks). The tanks were located in central locations of large racks within the respective species rooms, ensuring that all tanks received similar illumination and were surrounded by other tanks on the sides. No enrichment was provided. Fish were fed ad libitum each afternoon at the end of the experiments (sticklebacks: defrosted bloodworm, zebrafish: Zebrafeed (Sparos)). No fish of either species died during these experiments. All animals were killed by a Schedule 1 procedure at the end of this study.

### Experimental procedure

In order to elucidate which part of the DNA collection method triggers changes to stress axis activity, behaviour or welfare, seven different experimental groups were investigated in both species: Non-manipulated (controls, group 1); netted underwater for 15 s and then released (group 2); netted, restrained in a net out of water for 15 s and then released (group 3); netted, restrained in a net out of water, swabbed and then released (group 4); netted, anaesthetised with MS-222, restrained in a net out of water and allowed to recover (group 5); netted, anaesthetised, restrained in a net out of water, swabbed and allowed to recover (group 6); and netted, anaesthetised, restrained in a net out of water, fin clipped and allowed to recover (group 7). The components of each sampling method are summarised in Table 1. Skin swabbing was carried out as described in Breacker et al.^28^. Mucus samples were collected by gently stroking a sterile rayon-tipped swab five times along the flank of each fish, from the operculum to the base of the caudal fin. Very little pressure was used to collect mucus to avoid damaging the animal. Fins were clipped using a sterile razor blade, taking care to only remove about one third of the caudal fin. All fin clips and skin swabs were carried out by the same researcher to minimise any differences in handling the animals.

**Table 1:** Manipulations applied to each of the seven experimental groups.

Manipulation	Non-manipulated	Netted	Held out of water	Anaesthetised	Swabbed	Fin clipped	Released/recovery
Group 1 (control)	✓						
Group 2		✓					✓
Group 3		✓	✓				✓
Group 4		✓	✓		✓		✓
Group 5		✓	✓	✓			✓
Group 6		✓	✓	✓	✓		✓
Group 7		✓	✓	✓		✓	✓

#### Three-spined sticklebacks

Experiments were conducted inside the fish facility because it was large enough to allow behavioural recordings to take place at a suitable distance from the racks. For each manipulation the 13.4 L housing tanks containing the 27 fish were taken out of the system and placed onto a trolley 30 min before the experiments began.

#### Zebrafish

Experiments were conducted in a dedicated behaviour room which was maintained at the same temperature as the aquarium facility. The 3.5 L housing tanks containing the 30 fish designated for each manipulation were taken out of the system, placed onto a trolley, moved into the room where the recordings took place and left for 30 min before the experiments began. The distance moved was around 5 m.

Behaviour was recorded between 10:00 and 16:00. The experiments were independently repeated 3 times using the conditions detailed below. Immediately after manipulation (netting, swabbing, fin clipping etc.: Table 1) 12 fish were used to record the opercular beat rate (OBR). These fish were then kept in a 3.5 L holding tank for 2 h before the remaining behaviour tests (novel tank diving, open field and black and white preference) were carried out. Behaviour tests (not including OBR) were repeated at 24 h and 7 days post manipulation. One hour after the last manipulation 3 fish were used for cortisol measurements. Two hours after manipulation 4 stickleback and 5 zebrafish that were not used for behavioural testing were dissected to collect brain tissue for gene expression analysis. The dissected brains were snap frozen for RNA extraction at a later date. Similar dissections were also carried out at 24 h and 7 days post manipulation. The fish used for behavioural tests were returned to their home tanks for a further 21 days before being processed for body indices as a measure of long term effects on health and welfare.

### Water-borne cortisol measurements

#### Water sample collection

Cortisol measurements were made following protocols from Ellis et al.^23^, adapted by Sebire et al.^38,39^. One hour post manipulation 3 fish from each group were placed into individual 200 ml glass beakers containing 100 ml of freshly prepared reverse osmosis water. This water contained Instant Ocean salts (Aquarium Systems, UK) ensuring the same conductivity as the system water without background cortisol traces. The fish were left undisturbed for 30 min to allow cortisol release into the water. The beakers were separated by white dividers to prevent fish from seeing each other during this period. After 30 min the 100 ml water sample was collected using a serological pipette and split into two 50 ml Falcon tubes. Blank samples of 100 ml of RO water were also collected each week during the experimental period. 0.5 ml of methanol was added to each water sample prior to snap freezing in dry ice and storage at − 80 °C for extraction at a later date. The fish were euthanised by a lethal overdose of MS-222 and their dry weight was recorded to within 0.0001 g in order to calculate the cortisol concentration expressed as ng cortisol/g fish per h.

#### Cortisol extraction and quantification

Cortisol was extracted from the water samples by pumping it through Sep-pak Plus C18 solid phase extraction cartridges (Waters Ltd., UK) following the protocol developed by the Cefas Weymouth Laboratory^23^. Cartridges were primed with 5 ml of methanol followed by 5 ml of distilled water (dH_2_0) and water samples were pumped through the cartridges at 5 ml/min. Each cartridge was washed with 5 ml of dH_2_0, then air-dried, wrapped in Parafilm® and stored at − 80 °C until elution with 5 ml ethyl acetate. For the quantification by radioimmunoassay (RIA)^23,38,40^, the eluded extracts were evaporated at 45 °C under nitrogen and each residue was reconstituted in 500 µl of RIA buffer until assayed. The elution and the quantification were carried out in a blind manner in terms of the associated groups i.e. the species identity was known but not which manipulation the fish have undertaken.

### Behavioural methods

Behaviour was recorded using FlyCapture2 2.5.2.3 software and two digital cameras from Point Grey Research. The cameras were connected to a PC (Hewlett Packard) which was used to record videos in Audio Video Interleave format. Ethovision XT12 software (Noldus) was used for video tracking^41^. Some behavioural read-outs had to be scored manually since videotracking was not possible on the dark side of the black/white aquarium: freezing behaviour, time spent in the white zone and the number of times the fish crossed the boundary between black and white zones. These were scored by replaying the videos in Windows Media Player (Microsoft). To avoid any bias, the videos were renamed and manually scored in a blind manner. We conducted the following experiments:

### Opercular beating rate (OBR)

We tested OBR immediately after manipulation by placing single fish in small 1 L plastic tanks (12.5 × 7.5 × 12 cm) surrounded by white opaque material and recorded from above for 1 min. We recorded three tanks simultaneously. After four rounds of recordings, the 12 fish tested for OBR were kept in an intermediate tank placed on the same trolley (13.4 L for three-spined sticklebacks, 3.5 L for zebrafish) for 2 h until the battery of behavioural tests started. Analysis of the videos was carried out by two researchers blind to the identity of each fish. The OBR was manually quantified for the first 30 s of films by replaying the videos at reduced speed and the result was expressed as beats per minute.

### Novel tank diving test (NTT)

Two hours after recording the OBR we performed the novel tank diving test NTT to measure anxiety-like behaviour^42^. Sticklebacks were tested in standard 3.5 L trapezoid tanks (27.9 × 22.5 × 11.5 × 15 cm, L (top) × L (bottom) × W × H)^41^. Zebrafish were tested in a narrower tank (27.9 × 22.5 × 4.5 × 15 cm, L (top) × L (bottom) × W × H). Single fish were placed in this setup and recorded from the side for 5 min^43^. Two tanks were recorded simultaneously. The tank was divided in three zones—bottom, middle and top—for the analysis of the videos in Ethovision. We automatically measured the amount of time spent in the top third of the tank and total distance swum. We manually quantified freezing as cessation of swimming for more than 10 s while at the bottom of the tank^43^.

### Open field followed by black-white preference test

Immediately after performing the NTT the fish were gently transferred into the tanks used for recording general locomotion and exploratory activity followed by the black-white preference test^44^. This setup consisted of a transparent plastic tank (30 × 20 × 12 cm, L × W × H). The water depth was 10 cm. Single fish were placed in the centre of the tank, allowed to acclimate for 1 min and recorded from above for 5 min. When the first videos had been acquired we manually moved two removable opaque plastic covers that divided the tank into a black and white zone^41^ and recorded from above for further 5 min. Two tanks were recorded in parallel. To investigate locomotion and exploratory activity we used Ethovision to measure the total distance swum, and time spent in both the centre of the tank (an area half the size of the tank) and in the periphery swimming close to the walls. We manually quantified the total time spent in the white zone and the number of crosses from the black to the white zone. We considered a cross when the pectoral fins were in the white half of the tank. After the black-white preference test the fish were transferred into their housing tank^44^. When the 12 fish used to measure the OBR had undergone this battery of tests the home tank was placed back into the rack, connected to the system and fed. The same tests were performed using the same groups of fish 24 h and 7 days later.

### Gene expression analysis

We investigated five genes to examine the activation of the stress response by skin swabbing or fin clipping: *brain-derived neurotrophic factor* (*bdnf*)^45^, *corticotropin releasing hormone a* (*crha*)*, corticotropin releasing hormone b* (*crhb*)^46^, *galanin* (*galn*)^47^ and *neuropeptide y* (*npy*)^48^.

#### RNA extraction

Total RNA was extracted from frozen brain tissue using the GenElute Mammalian Total RNA Miniprep Kit (Sigma-Aldrich, UK) with a final elution volume of 30 µl^43^. The extracted RNA was treated with TURBO DNase (ThermoFisher Scientific, USA) to remove any genomic DNA. The concentration and purity of the RNA was determined using a NanoDrop 2000 spectrophotometer (LabTech International, UK) and 3 µL of total RNA was electrophoresed on a non-denaturing 1.5% (w/v) agarose gel to check for degradation.

#### First strand cDNA synthesis

First strand cDNA was reverse transcribed from total RNA (sticklebacks: 0.5 µg; zebrafish 0.125 µg) using a RevertAid First Strand cDNA Synthesis Kit (ThermoFisher Scientific, USA) following the manufacturer’s instructions. We used a mix of both oligo dT and random hexamers in a 20 μl reaction followed by a 1:4 dilution for stickleback samples and a 1:2 dilution for zebrafish with ddH_2_0.

#### Reverse-transcription quantitative PCR (RT-qPCR)

Reverse-transcription quantitative PCR (RT-qPCR) analysis was performed to examine marker gene expression, with primers designed to amplify 70–120 bp PCR products (primer details shown in Table 2). The RT-qPCR mixture consisted of 10 µL SensiFAST SYBR No-ROX Kit (Bioline, UK), 250 nM of forward and reverse primers (Table 2), 1 µL diluted cDNA and sterile water up to a total volume of 20 µL^43^. The RT-qPCRs were performed in triplicate on a CFX Connect qPCR thermocycler (BioRad Laboratories, CA) with the following cycling parameters: 95 °C for 3 min, followed by 40 cycles of 95 °C for 30 s, 60 °C for 30 s, and 72 °C for 30 s. A melting curve step (50–95 °C) was then performed, to verify that only single products had been amplified. No-template (NTCs) and no-reverse transcriptase controls (NRTs) were included for each primer pair and cDNA sample, respectively. Dilution series for each primer were performed to calculate amplification efficiency. This was calculated as: PCR efficiency (%) = (10(–1/S) − 1) × 100 where S = the slope of the standard curve from plotting the C_T_ values against the log template amount.

**Table 2:** qPCR primers manufactured by Sigma-Aldrich (Dorset, UK).

Gene	Forward primer	Reverse primer	Reference
**Stickleback**			
* bdnf*	5′-GACCAAGGATGTCGACCTGT-3’	5′-GCTGTCACCCACTGGCTAAT-3’	Lai, et al.^70^
* crha*	5′-GATCTGACCTTCCACCTGCTGAGA-3’	5′-GGTGTCCATCATCTTGCGGTTG-3’	Primer3 (designed)
* crhb*	5′-CGCCAAAGATCTCCGTTTAG-3’	5′-CCGTATACGCGCCATAGTTT-3’	Primer3 (designed)
* galn*	5′-GATGGAGACGTCATCCACACCATC-3’	5′-CATCTGATGTCACAGAGGACCGGC-3’	Primer3 (designed)
* npy*	5′-GAGGCACTACATCAACCTCATCA-3’	5′-GCTTTCCTTCAACAGCAGCTCTG-3’	Primer3 (designed)

Reference	Forward primer	Reverse primer	
* rpL8*	5′-CGACCCGTACCGCTTCAAGAA-3’	5′-GGACATTGCCAATGTTCAGCTGA-3’	Geoghegan et al.^71^
* rpL13A*	5′-CACCTTGGTCAACTTGAACAGTG-3’	5′-TCCCTCCGCCCTACGAC-3’	Hibbeler et al.^72^
* ubiq*	5′-AGACGGGCATAGCACTTGC-3’	5′-CAGGACAAGGAAGGCATCC-3’	Hibbeler et al.^72^

Gene	Forward primer	Reverse primer	Reference
**Zebrafish**			
* bdnf*	5′-CCTTACCATGGATAGCAAAAGGAA-3’	5′-CTATCTGCCCCTCTTAATGGTCAA-3’	Primer3 (designed)
* crha*	5′-CAGCAGACTCTCACCGACAA-3’	5′-ACACCGCAACGACAACCA-3’	Primer3 (designed)
* crhb*	5′-CATCCCAGTATCCAAAAAGAGC-3’	5′-TCGTAGCAGATGAAAGGTCAGA-3’	Sarath Babu et al.^73^
* galn*	5′-GACCAACTGATACTCAGGATGCA-3’	5′-ATCCCGAGTGTTTCTGTCAGAA-3’	Podlasz et al.^74^
* npy*	5′-GACTCTCACAGAAGGGTATCC-3’	5′-GGTTGATGTAGTGTCTTAGTGCTG-3’	Yokobori et al.^75^

Reference	Forward primer	Reverse primer	
* rpl13*	5′-TCTGGAGGACTGTAAGAGGTATGC-3	5′-AGACGCACAATCTTGAGAGCAG-3’	Primer3 (designed)
* elf1a*	5′-CCTCTTGGTCGCTTTGC-3’	5′-GGTGTGATTGAGGGAAATTCA-3’	Primer3 (designed)

The data was normalised against the geometric means of *ribosomal protein L8 (rpL8)*, *ribosomal protein L13A (rpL13A)* and *ubiquitin* (*ubiq*) genes in sticklebacks and *ribosomal protein L13A (rpL13A)* and *elongation factor 1a* (*elf1a*) genes in zebrafish. The fold change was calculated using the 2^−ddCT^ method^49^. The dCT value for each sample was determined by calculating the difference between the cycle time (CT) value of the gene of interest (GOI) and the CT value of the reference (REF) gene. This was determined for each unknown sample (sample) as well as for the control sample (calibrator). dCT (sample) = CT _(sample GOI)_ − CT _(sample REF)_; dCT (calibrator) = CT _(calibrator GOI)_ − CT _(calibrator REF)_. The ddCT value for each sample was determined by subtracting the dCT value of the calibrator from the dCT value of the sample. ddCT = dCT (sample) − dCT (calibrator). The fold change of the normalized level of GOI expression was calculated by using the formula: 2^-ddCT^.

### Long-term health and condition indicators

A number of body condition indices were calculated to determine whether DNA sampling has long term effects on fish. Twenty-eight days post manipulation the 12 fish that had been used in the behavioural studies were euthanised by a lethal dose of MS-222 anaesthetic. They were blotted dry, weighed (to 0.0001 g) and dissected. The spleen, liver, kidney and gonads were removed and weighed (to 0.0001 g). The following indices were calculated: Hepatosomatic Index (I_L_) = ([Liver Weight/Fish weight] × 100); Splenosomatic Index (I_S_) = ([Spleen Weight/Fish weight] × 100); Gonadosomatic Index (I_G_) = ([Gonad Weight/Fish weight] × 100); Nephrosomatic Index (also known as kidneysomatic index^50^), male sticklebacks only (I_K_) = ([Kidney Weight/Fish weight] × 100).

### Statistical analysis

Statistical analyses were carried out using GraphPad Prism7. Data were tested for normality using the using the Shapiro–Wilk test. Since the majority of data were not distributed normally, we analysed all data using the non-parametric Kruskal–Wallis test followed by a Dunn's multiple comparisons test comparing each treatment to the control group. Variation in data spread was analysed using The R package cvequality Version 0.1.3;^51^. We carried out an asymptotic test for the equality of coefficients of variation from k populations^52^. In this case we compared fish that had been swabbed without anaesthetic or fin clipped to control (undisturbed) fish. Figure 7 and all tables were prepared in Excel (Microsoft).

## Results

We measured changes in gene expression, behaviour and condition indicators at various time points after manipulation to investigate whether skin swabbing represents a less invasive method of collecting DNA from live fish than fin clipping. We recorded each variable in seven groups of sticklebacks or zebrafish (Table 1). Comparing these different treatment groups allowed us to separate the influence of netting, anaesthesia, skin swabbing and fin clipping on subsequent observations.

### Early indicators of stress activation

**Figure 1:**
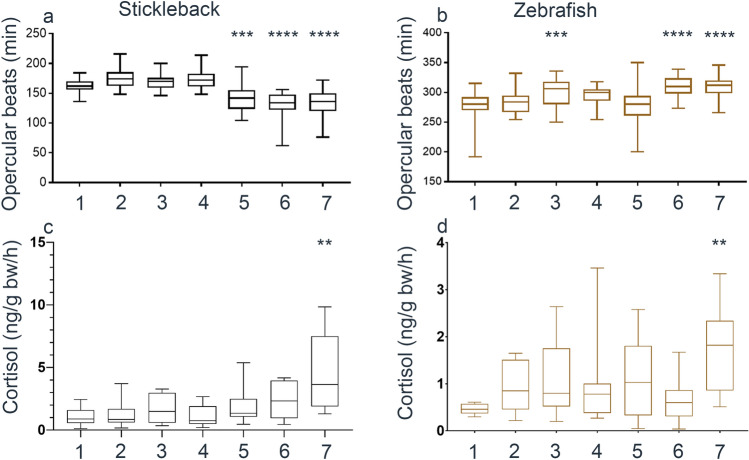
Early indicators of stress activation. (**a**, **b**) Changes to number of opercular beats per minute and (**c**, **d**) cortisol excretion in stickleback (**a**, **c**) and zebrafish (**b**, **d**). Manipulation groups: 1 undisturbed; 2 netted under water; 3 netted air; 4 swabbed; 5 MS-222; 6 MS-222 and swabbed; 7 MS-222 and fin clipped. Letters on graphs not shared in common between groups indicate significant differences.

We first measured two early indicators of stress: opercular beat rate (OBR; an indicator of ventilation response of fish to stress^53^ and metabolic rate^54^); and excretion of the stress hormone cortisol into water^23,38^ (Table S1). Groups of sticklebacks treated with MS-222 displayed reduced OBR compared to the other manipulations, suggesting that immersion in anaesthetic decreased ventilation (Fig. 1a). The release of cortisol into water was significantly increased by fin clipping but no other manipulation (Fig. 1c). In zebrafish, OBR was increased in the group netted in air, and in fish that were anaesthetised and swabbed or fin clipped (Fig. 1b). Similar to sticklebacks, cortisol release into water was only significantly increased following fin clipping, although at a lower level (Fig. 1d). Taken together, these results suggest that anaesthetic treatment affects OBR, albeit in a different direction across species; and that fin clipping is more stressful than any other treatment when using cortisol excretion as a read-out.

### Changes to behaviour after swabbing or fin clipping

**Figure 2:**
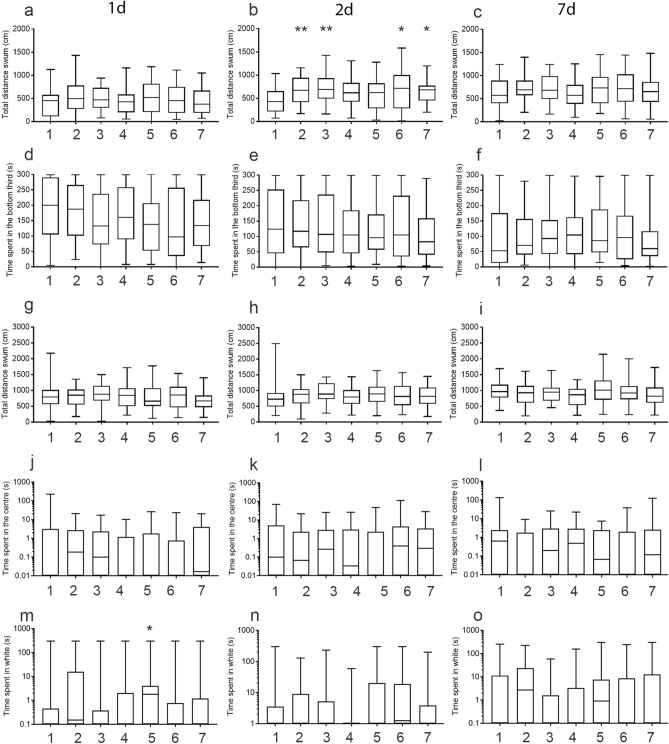
Long-term behavioural alterations in stickleback. (a) Distance swum in novel tank diving test, day 1. (**b**) Distance swum in novel tank diving test, day 2. (**c**) Distance swum in novel tank diving test, day 7. (**d**) Time in bottom of novel tank, day 1. (**e**) Time in bottom of novel tank, day 2. (**f**) Time in bottom of novel tank, day 7. (**g**) Distance swum in open field test, day 1. (**h**) Distance swum in open field test, day 2. (**i**) Distance swum in open field test, day 7. (**j**) Time in centre of open field tank, day 1. (**k**) Time in centre of open field tank, day 2. (**l**) Time in centre of open field tank, day 7. (**m**) Time in white half of black/white tank, day 1. (**n**) Time in white half of black/white tank, day 2. (**o**) Time in white half of black/white tank, day 7. Manipulation groups: 1 undisturbed; 2 netted under water; 3 netted air; 4 swabbed; 5 MS-222; 6 MS-222 and swabbed; 7 MS-222 and fin clipped. The Y axis is log-transformed in (**j**)–(**o**) to better depict data. All statistical analyses were performed on raw (untransformed data).

We next considered the medium-term impact of swabbing and fin clipping on behaviour one day, two days and seven days after manipulation. We compared different tests for anxiety-like behaviour including the novel tank diving test^42^ (Table S2), open field test^55^ and light/dark box^56^ (Table S3). On day 1, sticklebacks displayed no difference in the distance swum in the novel tank regardless of treatment group (Fig. 2a). On day 2, the groups that were netted underwater, held in a net in the air, or treated with MS-222 and either swabbed or fin clipped all swam further than the other groups (Fig. 2b). This suggests that both netting and anaesthetic treatment had a delayed impact on locomotion. This effect disappeared by day 7, with no changes to the distance swum in any of the groups (Fig. 2c). The time spent at the bottom of the tank, a measure of anxiety-like behaviour^42^, was similar across treatments at all time points (Fig. 2d–f). We further investigated anxiety in the open field and light/dark tests. In contrast to our previous findings, sticklebacks showed no alterations in locomotion (Fig. 2g–i) or time in the centre of the open field (Fig. 2j–l), indicating that DNA collection did not affect anxiety in this test. In the light/dark test sticklebacks that were anaesthetised without being clipped or swabbed spent significantly more time in the white zone of the light/dark box (indicating reduced anxiety) on day 1 (Fig. 2m). There were no differences between treatment groups on days 2 (Fig. 2n) or 7 (Fig. 2o).

**Figure 3:**
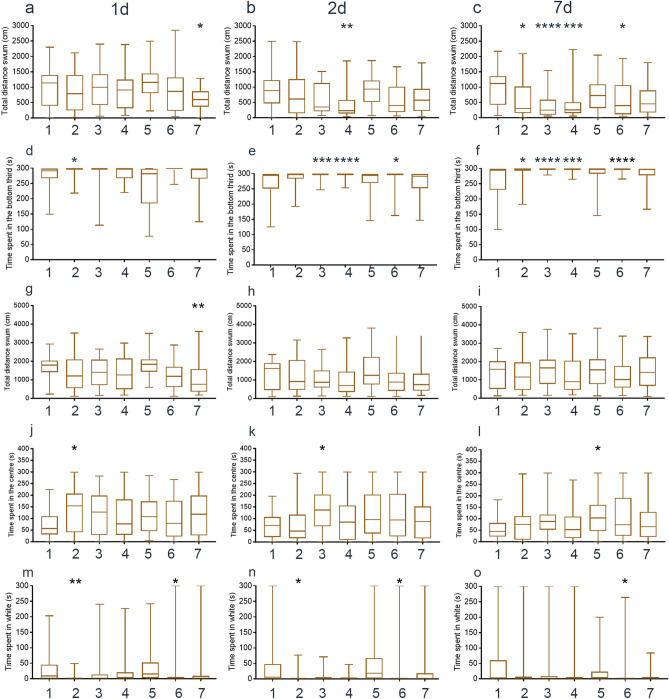
Long-term behavioural alterations in zebrafish. (**a**) Distance swum in novel tank diving test, day 1. (**b**) Distance swum in novel tank diving test, day 2. (**c**) Distance swum in novel tank diving test, day 7. (**d**) Time in bottom of novel tank, day 1. (**e**) Time in bottom of novel tank, day 2. (**f**) Time in bottom of novel tank, day 7. (**g**) Distance swum in open field test, day 1. (**h**) Distance swum in open field test, day 2. (**i**) Distance swum in open field test, day 7. (**j**) Time in centre of open field tank, day 1. (**k**) Time in centre of open field tank, day 2. (**l**) Time in centre of open field tank, day 7. (**m**) Time in white half of black/white tank, day 1. (**n**) Time in white half of black/white tank, day 2. (**o**) Time in white half of black/white tank, day 7. Manipulation groups: 1 undisturbed; 2 netted under water; 3 netted air; 4 swabbed; 5 MS-222; 6 MS-222 and swabbed; 7 MS-222 and fin clipped. The Y axis is log-transformed in (**j**)–(**o**) to better depict data. All statistical analyses were performed on raw (untransformed data).

Zebrafish displayed long-term alterations to anxiety-like behaviour in the novel tank diving (Table S4), open field and light/dark tests (Table S5) after DNA collection. The total distance swum in the novel tank was decreased in fin clipped zebrafish on day 1 (Fig. 3a). On day 2, the distance swum was decreased in zebrafish that had been swabbed without anaesthetic (Fig. 3b), whereas on day 7 it was decreased in the groups that had been netted underwater, held in a net suspended in air, swabbed without anaesthetic or swabbed after MS-222 treatment, (Fig. 3c) indicating a long-term change in swimming after DNA collection in this species. The amount of time spent at the bottom of the novel tank also varied over time. Zebrafish that were netted and held under water spent more time at the bottom of the tank on day 1 (Fig. 3d). On day 2, zebrafish that were held in air, and swabbed with- or without anaesthetic spent more time at the bottom of the novel tank (Fig. 3e). A similar result was seen on day 7, with groups of zebrafish that were netted underwater, held in a net suspended in air, and swabbed with- or without anaesthetic spending more time at the bottom of the tank (Fig. 3f). This suggests that swabbing increases anxiety-like behaviour in this test, with a stronger effect over time. In the open field test, fin clipping led to a reduction in the total distance swum on day 1 (Fig. 3g) with no other differences between groups on days 2 and 7 (Fig. 3h,i). Holding in a net under water increased the amount of time that zebrafish spent in the centre of the open tank on day 1 (Fig. 3j). Netting and holding in air had a similar effect on day 2 (Fig. 3k) and anaesthetising zebrafish led to more time being spent in the centre on day 7 (Fig. 3l). In the light/dark test, both holding zebrafish in a net underwater and swabbing them after applying MS-222 decreased the time spent on the white side, a readout of anxiety-like behaviour, on day 1 (Fig. 3m) and day 2 (Fig. 3n). This effect was maintained in the swabbed and anaesthetised group on day 7 (Fig. 3o).

### Expression of stress marker genes after skin swabbing or fin clipping

**Figure 4:**
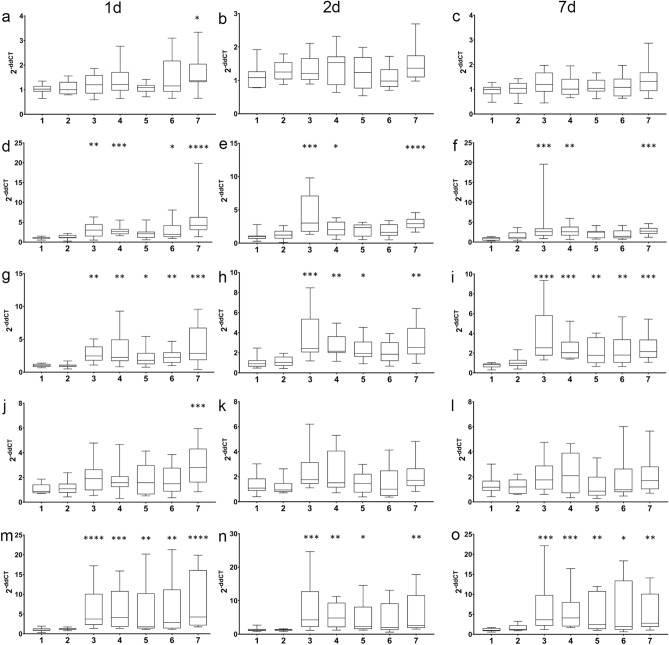
Expression of stress marker genes in stickleback. qPCR data showing expression of (**a**) *brain-derived neurotrophic factor* (*bdnf*) day 1. (**b**) *brain-derived neurotrophic factor*, day 2. (**c**) *brain-derived neurotrophic factor*, day 7. (**d**) *corticotropin releasing hormone a* (*crha*) day 1. (**e**) *corticotropin releasing hormone a*, day 2. (**f**) *corticotropin releasing hormone a*, day 7. (**g**) *corticotropin releasing hormone b* (*crhb*) day 1. (**h**) *corticotropin releasing hormone b*, day 2. (**i**) *corticotropin releasing hormone b*, day 7. (**j**) *galanin* (*galn*) day 1. (**k**) *galanin* day 2. (**l**) *galanin* day 7. (**m**) *neuropeptide y* (*npy*) day 1. (**n**) *neuropeptide y* day 2. (**o**) *neuropeptide y* day 7. Manipulation groups: 1 undisturbed; 2 netted under water; 3 netted air; 4 swabbed; 5 MS-222; 6 MS-222 and swabbed; 7 MS-222 and fin clipped.

To examine the longer term impact of swabbing and fin clipping on stress response we used quantitative PCR to compare the expression level of genes related to the stress response: *brain-derived neurotrophic factor* (*bdnf*)^45^, *corticotropin releasing hormone a* (*crha*), *corticotropin releasing hormone b* (*crhb*)^46^, *galanin* (*galn*)^47^ and *neuropeptide y* (*npy*)^48^ over time. No amplification was recorded from the qPCR control cDNA samples (NTCs or NRTs). In stickleback, *bdnf* expression was altered by fin clipping on day 1, but not day 2 or 7 (Fig. 4a–c; Table S6). *crha* expression was increased in sticklebacks that had been netted in air, swabbed without anaesthetic, swabbed with anaesthetic or fin clipped on day 1 (Fig. 4d). On day 2, *crha* expression was elevated in sticklebacks that had been netted in air, swabbed without anaesthetic or fin clipped (Fig. 4e), and a similar result was seen on day 7 (Fig. 4f). *crhb* expression was increased in the netted in air, swabbed without anaesthetic and treated with MS-222 groups on day 1 (Fig. 4g). A similar pattern was seen on days 2 (Fig. 4h) and 7 (Fig. 4i), although the sticklebacks fin clipped with anaesthetic recovered on day 2 but not day 7. The expression of *galn* was increased in fin clipped sticklebacks compared to all other groups on day 1 (Fig. 4j). There were no differences in expression at the other time points measured (Fig. 4k,l). Finally, *npy* expression was increased in fish netted in air, swabbed with- or without anaesthetic, treated with anaesthetic alone or fin clipped on day 1 (Fig. 4m). This effect was maintained in sticklebacks netted in air, swabbed without anaesthetic, treated with anaesthetic alone or fin clipped on day 2 (Fig. 4n), whereas on day 7 all groups of fish apart from those netted underwater displayed heightened *npy* expression (Fig. 4o).

**Figure 5:**
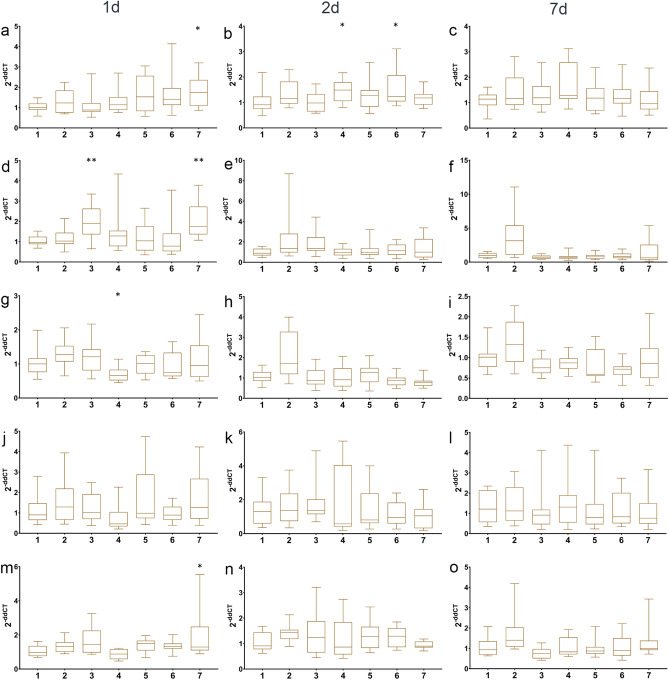
Expression of stress marker genes in zebrafish. qPCR data showing expression of (**a**) *brain-derived neurotrophic factor* (*bdnf*) day 1. (**b**) *brain-derived neurotrophic factor*, day 2. (**c**) *brain-derived neurotrophic factor*, day 7. (**d**) *corticotropin releasing hormone a* (*crha*) day 1. (**e**) *corticotropin releasing hormone a*, day 2. (**f**) *corticotropin releasing hormone a*, day 7. (**g**) *corticotropin releasing hormone b* (*crhb*) day 1. (**h**) *corticotropin releasing hormone b*, day 2. (**i**) *corticotropin releasing hormone b*, day 7. (**j**) *galanin* (*galn*) day 1. (**k**) *galanin* day 2. (**l**) *galanin* day 7. (**m**) *neuropeptide y* (*npy*) day 1. (**n**) *neuropeptide y* day 2. (**o**) *neuropeptide y* day 7. Manipulation groups: 1 undisturbed; 2 netted under water; 3 netted air; 4 swabbed; 5 MS-222; 6 MS-222 and swabbed; 7 MS-222 and fin clipped.

Swabbing or fin clipping zebrafish led to activation of fewer stress axis marker genes (Table S7). Fin clipping led to an increase in *bdnf* expression on day 1 (Fig. 5a). On day 2, heightened *bdnf* expression was seen in zebrafish that were swabbed with or without anaesthetic (Fig. 5b). On day 7, there were no significant changes in *bdnf* expression compared to control animals (Fig. 5c). Fin clipping and netting in air altered the expression of *crha* on day 1 (Fig. 5d). No significant changes in expression were seen on days 2 or 7 (Fig. 5e,f). *crhb* expression was decreased by swabbing on day 1 (Fig. 5g), but no other changes were seen at the other time points analysed (Fig. 5h,i). There were no differences in *galn* expression seen in any manipulation group over time (Fig. 5j–l). *npy* expression was activated by fin clipping on day 1 (Fig. 5m). However, no changes in expression were seen in any treatment group over time (Fig. 5n,o).

### Changes to long-term health and condition indicators

**Figure 6:**
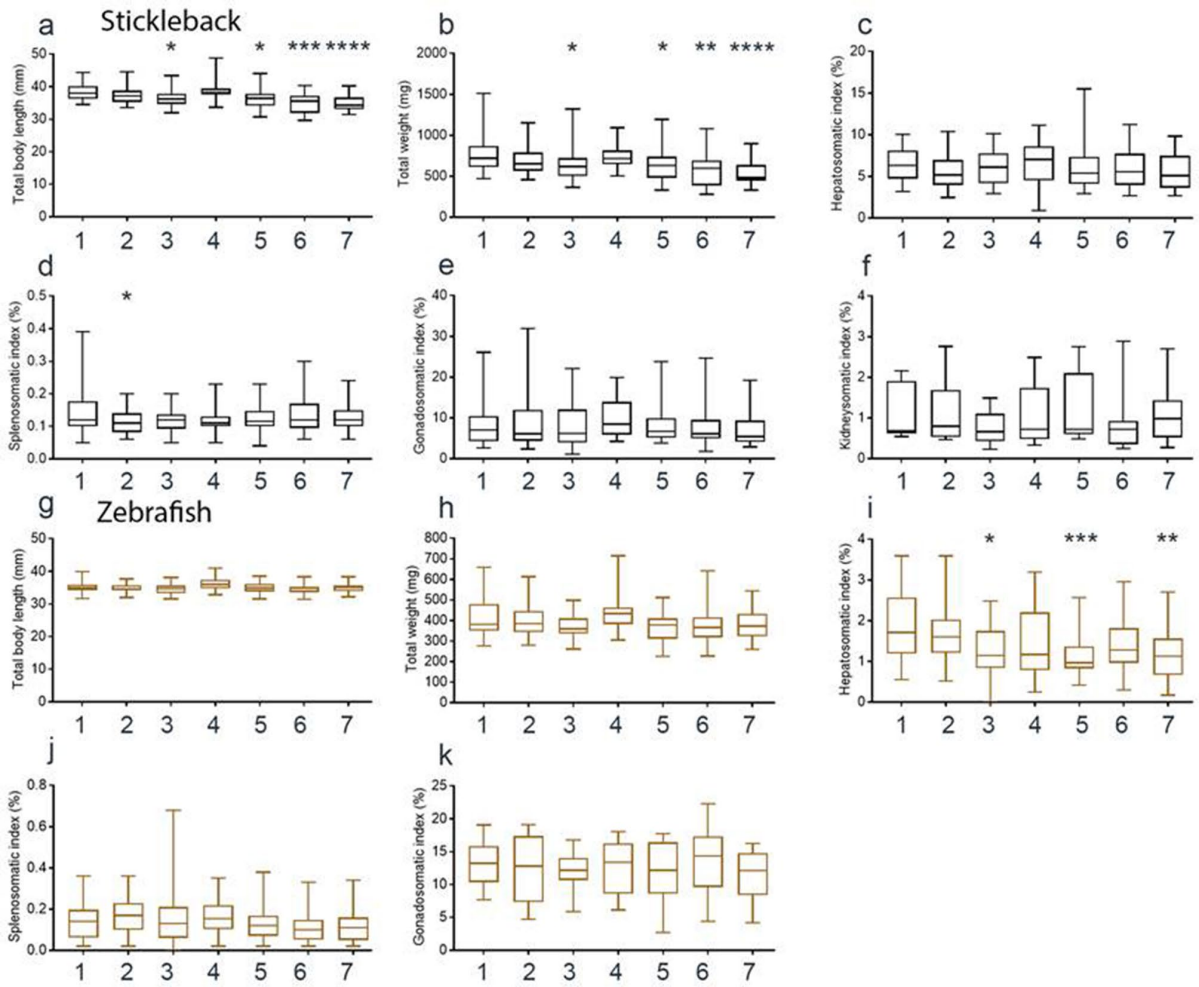
Health and condition indicators in stickleback and zebrafish. Growth and health indicators 28 days after swabbing or fin clipping. (**a**) Stickleback body length. (**b**) Stickleback body weight. (**c**) Stickleback hepatosomatic index. (**d**) Stickleback splenosomatic index. (**e**) Stickleback gonadosomatic index. (**f**) Stickleback nephrosomatic index. (**g**) Zebrafish body length. (**h**) Zebrafish body weight. (**i**) Zebrafish hepatosomatic index. (**j**) Zebrafish splenosomatic index. (**k**) Zebrafish gonadosomatic index. Manipulation groups: 1 undisturbed; 2 netted under water; 3 netted air; 4 swabbed; 5 MS-222; 6 MS-222 and swabbed; 7 MS-222 and fin clipped.

One month after manipulation, we investigated health and condition indicators in both stickleback and zebrafish, including body length and weight, and the hepatosomatic, splenosomatic, gonadosomatic and nephrosomatic indices (Table S8). The different indices show different aspects of fish health. A large liver can indicate a healthy fish^57^; fish displaying an immune response will have an increased spleen size^58^, and normal sexual development can be determined by the size of gonads in females^59^ and kidneys in male sticklebacks^60,61^. In sticklebacks, we recorded a decrease in body length (Fig. 6a) and weight (Fig. 6b) in fish that were held in air or treated with anaesthetic. There was a decrease in the splenosomatic index for sticklebacks netted under water (Fig. 6d), but no differences in the hepatosomatic (Fig. 6c), gonadosomatic (Fig. 6e) or nephrosomatic (Fig. 6f) indices. Neither skin swabbing nor fin clipping altered the body length or weight in zebrafish (Fig. 6g,h). There was a decreased hepatosomatic index in zebrafish that were held in air, treated with MS-222 or fin clipped (Fig. 6i). No changes to the splenosomatic or gonadosomatic indices were observed, regardless of manipulation (Fig. 6j,k).

## Discussion

**Figure 7:**
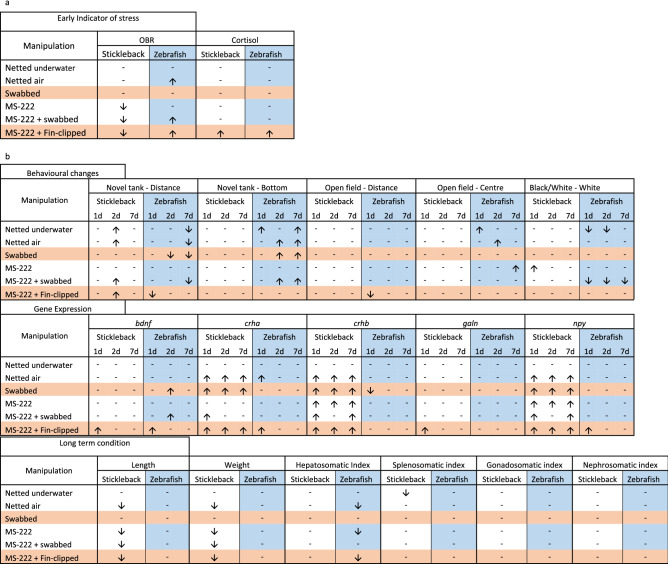
Summary of changes to stress axis activation, behaviour, gene expression and condition indicators following fin clipping or skin swabbing. Red shading indicates skin swabbing without anaesthetic (group 4) or fin clipping (group 7) for comparison with un-manipulated control animals (group 1). Arrow indicate increases (↑) or decreases (↓) of readouts, where a—indicates no change.

In this study we have compared two different methods to collect DNA from small species of fish that are regularly used in laboratory research; skin swabbing and fin clipping. Both techniques alter behaviour, stress axis activation and welfare when compared to control un-manipulated animals. In general, gene expression changes were more pronounced in sticklebacks than zebrafish following both procedures, whereas zebrafish displayed a greater number of alterations in behaviour compared to sticklebacks. However, skin swabbing appeared to have less impact than fin clipping in both species, even without the use of anaesthetic, since fewer of the read-outs that we measured were altered by this technique (Fig. 7a,b).

DNA collection by fin clipping caused a significant activation of the stress axis in both zebrafish and sticklebacks compared to control fish or swabbing without MS-222 treatment. This was measured by quantifying the amount of water-borne cortisol released in the first hour after the DNA sampling^38,62^. Since neither MS-222 treatment alone, nor MS-222 treatment followed by swabbing, altered cortisol release it appears that excision of the fin tissue causes this response. Our findings agree with some published studies but not others. For example, both application of MS-222 and fin clipping have been shown to increase cortisol release in zebrafish^19^. Conversely De Lombaert et al.^3^ reported no effect of fin clipping on whole-body cortisol levels. These different findings might be explained by the experimental protocol used. In our study we placed single fish into a beaker and sampled the amount of cortisol excreted during 30 min one hour after manipulation. White et al.^19^ also measured water-borne cortisol, but they used a siphon to sample the average release from a group of animals, whereas in the De Lombaert et al.^3^ study cortisol was extracted from the whole body, representing protein-bound levels of this hormone^3,19^. This suggests that cortisol excretion into water could be more sensitive than changes in whole-body cortisol to these manipulations, even though previous research has shown a positive correlation between both readouts^38,62^.

Although it is tempting to compare differences in behaviour between these two species, both slight differences in the experimental design (including transfer of zebrafish to another room before measuring behaviour) and the amount of time that each species was maintained in the lab before testing (several generations for zebrafish, compared to around one year for sticklebacks) complicates interpretation of our results. Nevertheless, fin clipping and swabbing triggered complex changes in behaviour that differed both between species and over time. After fin clipping sticklebacks displayed decreased opercular beat rate (OBR; Fig. 1a), whereas zebrafish displayed increased OBR (Fig. 1b). In agreement with this, previous research has already shown that fin clipping increases zebrafish OBR^21^. The opposing effect on OBR suggests that this technique affects respiration differently in these fishes, perhaps due to the level of anaesthesia obtained following immersion in MS-222; zebrafish may have been less sedated than sticklebacks leading a quickening of their ventilation.

Very few changes in behaviour were seen in either species in the novel tank, open field and black and white box tests. Sticklebacks displayed an increase in the distance swum in the novel tank 2 days after fin clipping, whereas swabbed sticklebacks did not. In zebrafish, fin clipping significantly decreased the distance swum in both the novel tank and open field arena on day 1 compared to swabbing without anaesthetic. This agrees with previous demonstrations that fin clipping heightens anxiety-like behaviour including reduced activity, increased time at the bottom of a tank and decreased feeding activity^3,6,19,21,22^*.* Our behavioural recordings were taken after 2 h, and our findings broadly agree with published studies that analysed behaviour after 1h^3,6,21,22^. However, there is ample evidence that the timing of measurement can affect the results obtained. For example, heightened anxiety-like behaviour recovered after 6 h in some studies but not others^3,21^. This could be due to the protocol used to collect tissue, including factors such as the amount of tissue that is removed from each animal.

Swabbing had a delayed effect on zebrafish anxiety-like behaviour, with changes seen on days 2 and 7, but not on day 1 (Fig. 3b,e). This could represent a complex response to DNA sampling involving a number of factors such as changes in immune response, ionic or osmotic regulation^31^. However, other non-swabbed groups also displayed changes to behaviour that were only present at one time point. For example, zebrafish that had been netted in air only spent more time in the centre of the open field tank (Fig. 3k) on day 2. Extrinsic factors such as changes in the time of feeding or tank cleaning in the fish facility could have contributed to the wide spread of these data points, both within each group and when comparing behaviour over time. It is hard to control for such factors when analysing data, highlighting the difficulty of comparing repeat measurements of behaviour over time.

Similar to the changes to behaviour noted above, qPCR measurements of stress axis marker genes show a complex profile of changes that differ across species. *bdnf* levels are decreased by stress in both Atlantic salmon^63^ and zebrafish^64^ suggesting a reduction in neural plasticity. *crha* and *crhb* code for Corticotropin-releasing hormone, which stimulates the hypothalamus-pituitary-interrenal axis to release cortisol. Both *crh* and *npy* are activated by the addition of cortisol to food in goldfish^65^, linking their expression to the stress response. *galn* expression has also been used as a general stress marker in zebrafish^47^). Sticklebacks displayed an increase in *bdnf* and *galn* expression on day 1 following fin clipping (Fig. 4a,j). *crha, crhb* and *npy* were altered by both methods at all time points assessed (Fig. 4d–i). In zebrafish, fin clipping increased *bdnf* and *npy* expression on day 1 (Fig. 5a, j) whereas swabbing led to heightened *bdnf* expression on day 2 (Fig. 5b). Interestingly, *crhb* expression was decreased by swabbing but not fin clipping in this species on day 1 (Fig. 5g). Together, these results reinforce the idea that whilst both methods are detrimental to fish, swabbing alters the expression of fewer genes suggesting that it may be less invasive than fin clipping. In some cases the impact of swabbing only appears on day 2 or 7, in keeping with the medium-term changes in behaviour seen above.

Neither skin swabbing nor fin clipping had much influence upon health and welfare indicators 1 month after manipulation. In sticklebacks, MS-222 treatment lead to fish being smaller and lighter (Fig. 4a,b), although the difference from other treatment groups was very small. In zebrafish, fin clipping decreased the hepatosomatic index suggesting that sexual maturation has been inhibited by this technique^57^. Another possibility is that energy storage is disrupted by fin clipping, which has been shown to trigger a short term decrease in feeding in zebrafish^3^. This could lead to a decreased liver size if maintained for a long period of time. In general, the absence of changes in health and condition indicators one month after DNA sampling suggests that removal of skin mucus or fin tissue does not have a long-term impact upon fitness in either species.

We found similar changes in the OBR, cortisol excretion, locomotion- and time at the bottom of the novel tank in both zebrafish and sticklebacks although the direction of change sometimes differed between species. This suggests that a combination of these four indicators could be used to assess the impact of manipulations upon fish in future studies, with a particular focus on short-term alterations within the first or second hour after DNA sampling. However, the wide variation in behaviour and gene expression that we observed, both across techniques and time, raises the question whether these measurements really indicate suffering in sticklebacks and zebrafish. One way to assess the preference of fish for either technique would be to use a conditioned place aversion test (e.g. Wong et al.^66^). We could first measure an animal’s baseline preference for either side of an unbiased two-colour tank. Fish would be allowed to recover in a single colour tank after performing either fin clipping or skin swabbing. In a final step we could re-measure place preference in the two-colour arena; if the DNA collection technique was stressful we might see an aversion to the paired colour from the recovery tank.

Another striking aspect of our data is the large amount of variability that we recorded, both within groups (including the un-manipulated control animals) and when comparing DNA collection methods. A wide intra-group variation is seen in the behaviour data (Figs. 2, 3). The wide spread of behavioural phenotypes could possibly represent different coping styles or personalities within the groups of animals that we tested^21,67^. DNA collection appears to interact with individual differences, with some swabbed or clipped animals showing similar behaviour and stress axis activity as undisturbed animals in both species. What governs the lack of reaction to DNA sampling in some animals is not known, but could include individual differences in stress axis reactivity, dominance status or the size of the fish. Further studies would be required to explore this phenomenon in more detail. For example, fish could be separated into groups of bold versus shy animals using the novel tank diving test^42^, interaction with a novel object^68^ or the time spent on the dark side of a black/white box^69^ before carrying out the fin clip or skin swabbing procedure. Regarding variation between treatment groups, fin clipping led to greater variation in experimental data compared to control treated animals vs. swabbing in both species (Table 3) when analysing OBR, cortisol and behaviour using the asymptotic test for the equality of coefficients of variation^52^. Since swabbing induces less variable (as well as less severe) stress responses in groups of zebrafish and sticklebacks, DNA collection by swabbing may permit the use of smaller sample sizes in experimental studies.

**Table 3:** Asymptotic test for the equality of coefficients of variation from k populations comparing physiological and behavioural data after skin swabbing or fin clipping.

Test name	Species	Control vs. clipped		Control vs. swabbed	
		Test statistic	*p* value	Test statistic	*p* value
OBR	Stickleback	56.96	**4.45E−14**	2.3	0.13
OBR	Zebrafish	75.35	**3.94E−18**	13.21	**0.00027**
Cortisol	Stickleback	15.4	**8.69E−05**	0.13	0.72
Cortisol	Zebrafish	− 0.31	0.57	5.64	**0.017**
Novel tank distance	Stickleback	50.49	**1.19E−12**	0.23	0.63
Novel tank distance	Zebrafish	63	**1.97E−15**	1.31	0.25
Novel tank time	Stickleback	71.17	**3.27E−17**	6.36	**0.01**
Novel tank time	Zebrafish	55.49	**9.40E−14**	0.02	0.88
Open field distance	Stickleback	64.31	**1.06E−15**	0.32	0.57
Open field distance	Zebrafish	60.57	**7.09E−15**	5.59	**0.02**
Open field time	Stickleback	7.35	**0.0067**	0.8	0.37
Open field time	Zebrafish	56.54	**5.49E−14**	0.35	0.55
Black white time	Stickleback	0.03	0.85	9.77	**0.001**
Black white time	Zebrafish	29.57	**5.38E−08**	0.24	0.62

Bold values indicate significant differences compared to control values.

In this study we have shown that skin swabbing is a refined technique to collect DNA from small fish species compared to fin clipping, the standard protocol used in most laboratories^18^. Skin swabbing triggers fewer changes in stress axis activation, behaviour and gene expression compared to fin clipping. It also leads to a smaller variation in physiological and behaviour data, with the potential to reduce the number of animals needed to collect statistically significant results. This may also aid in comparison of data across laboratories, thus helping to address one aspect of the reproducibility crisis in scientific research. We have already demonstrated that skin swabbing can be used to collect enough DNA for PCR amplification and fish identification, using zebrafish that are larger than 20 mm^28^. Skin swabbing is quicker, cheaper and safer than fin clipping because it does not require anaesthetic or scalpels to be used. It is simple to perform once researchers have been trained in the technique, although care must be taken to swab the fish from anterior to posterior using very light pressure to avoid activating nociceptors. Skin swabbing is also not currently considered to be a procedure under the Animals (Scientific Procedures) Act 1986 in the UK. In summary, swabbing is a more refined technique for DNA collection with the potential to have an extremely wide impact upon fish health and welfare. It may also reduce the number of animals required for some experiments.

## Supplementary information

Supplementary Information.
