# Supplementary material for: Skin swabbing is a refined technique to collect DNA from model fish species
Source: Sci Rep. 2020 Oct 23;10:18212. doi: 10.1038/s41598-020-75304-1 (PMC7584585; doi:10.1038/s41598-020-75304-1)
Supplement: Supplementary file 1 — Supplementary Information. [file 41598_2020_75304_MOESM1_ESM.docx]

**SKIN SWABBING IS A REFINED TECHNIQUE TO COLLECT DNA FROM MODEL FISH SPECIES**

Ceinwen A. Tilley^1^, Hector Carreño Gutierrez^1^, Marion Sebire^2^, Oluwapelumi Obasaju^1^, Florian Reichmann^1,3^, Ioanna Katsiadaki^2^, Iain Barber^4*^ and William H.J. Norton^1,5*^

1. Department of Neuroscience, Psychology and Behaviour, College of Life Sciences, University of Leicester, Leicester, LE1 7RH, UK

2. Cefas Weymouth Laboratory, Barrack Road, The Nothe, Weymouth, Dorset DT4 8UB, UK

3. Division of Pharmacology, Otto Loewi Research Centre for Vascular Biology, Immunology and Inflammation, Medical University of Graz, Graz, Austria

4. School of Animal, Rural & Environmental Sciences, Nottingham Trent University, Brackenhurst Campus, Brackenhurst Lane, Southwell NG25 0QF,UK

5. The International Zebrafish Neuroscience Research Consortium (ZNRC), Slidell, LA, USA

* Correspondence to Iain Barber, [iain.barber@ntu.ac.uk](mailto:iain.barber@ntu.ac.uk) and Will Norton, [whjn1@le.ac.uk](mailto:whjn1@le.ac.uk)

Keywords: Zebrafish, Stickleback, Fin clipping, Skin swabbing, behaviour, welfare, stress, cortisol, 3Rs

**Supplementary information**

Supplemental methods

Experimental design

Power analyses. Olfactory beat response (OBR) measurements: we had no previous data that we could use for power analysis. However, the other behavioural measurements required a maximum of 14 fish suggesting it is likely that n = 14 would be sufficient for this experiment. Cortisol release was based upon data comparing wild-type zebrafish to a *linc5* mutant^1^. Mean control = 20.0±3.9 pg/g, mean treatment group = 9.9±3.2 pg/g. (1-α) is 0.05 and power (1-β) is 80 giving an n value of 4. We used 9 animals since this fits better with three experimental replicates (i.e. 3 x 3 = 9). Behaviour data was based upon novel tank-diving data comparing wild-type zebrafish to a *nitric oxide 1* mutant^2^. Mean control = 186±73 s, mean treatment group = 294±11 s. (1- α) is 0.05 and power (1-β) is 80 giving an n value of 14. Locomotion (mean 25±5 s vs 19±3 s) and black/white tank data mean (105±26 s vs 44±21 s) from same study require a similar number of animals. qPCR numbers were based upon preliminary data: Control 33±0.8 and treatment 32±0.8. (1-α) is 0.05 and power (1-β) is 80 giving an n value of 8. We used 9 animals since this fitted better with three experimental replicates (i.e. 3 x 3 = 9). Health and condition indices were based upon pilot hepatosomatic index data from control and infected sticklebacks. Mean control 4.2±0.6 g vs 2.7±0.5 g (1-α) is 0.05 and power (1-β) is 80 giving sample size of 12. We measured 14 fish were also used for behaviour.

In vitro generation of sticklebacks

Male sticklebacks displaying classic nuptial colouration (red throats and blue eyes^3^) were selected and euthanized according to the UK Home Office approved Schedule 1 method. Testes were dissected and placed in an ice-cold watch glass and macerated in several drops of aquarium water. Gravid females were selected and their eggs were stripped into cooled watch glasses and covered with the solution from the macerated testes. After 15 min, eggs were checked for successful fertilisation by viewing the development of the outer membrane via a dissection microscope. Following confirmation of fertilisation, the eggs were transferred to 1 L (15.5 cm x 9.5 cm x 8.5 cm) plastic aquaria. Methylene Blue (2 ml of 1 mg/ml stock solution) was added to the aquarium water to prevent the development of fungal infections, and the water was aerated using an airstone. At 5 days post fertilisation, 75% of the water change was replaced, and then 50% of the water was changed each day until hatching to remove all Methylene Blue^4,5^. After hatching, fry were fed LiquidFry No1 (Interpet, Dorking, UK) for the first 5 days, before transferring to 2 day-old *Artemia* sp. nauplii fed daily, *ad libitum*, for three months.

Supplemental figures

Figure S1

Experimental design for cortisol sampling.


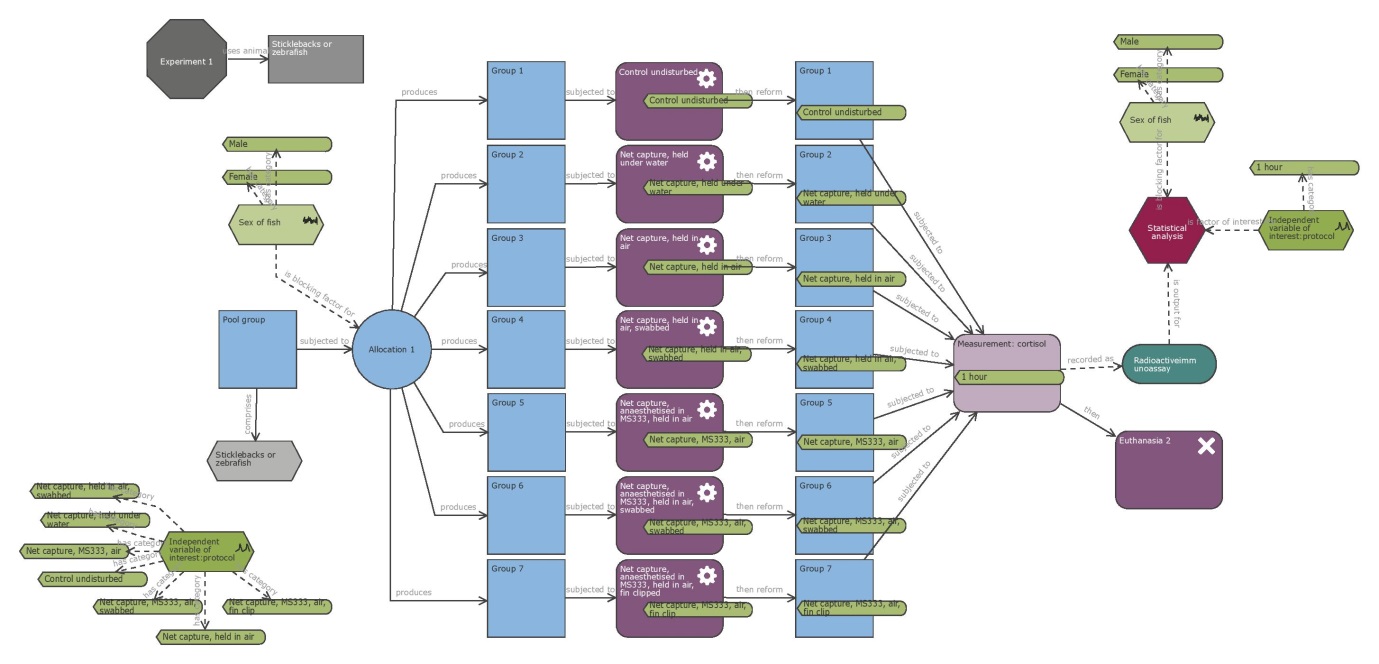


Figure S2

Experimental design for behavioural analysis


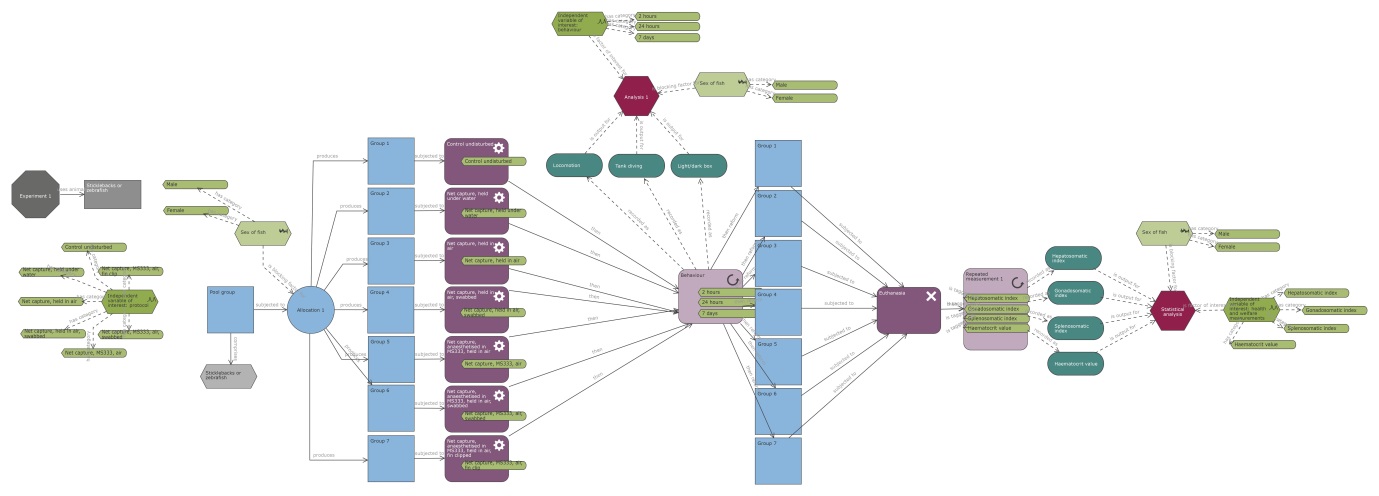


Figure S3

Experimental design for qPCR


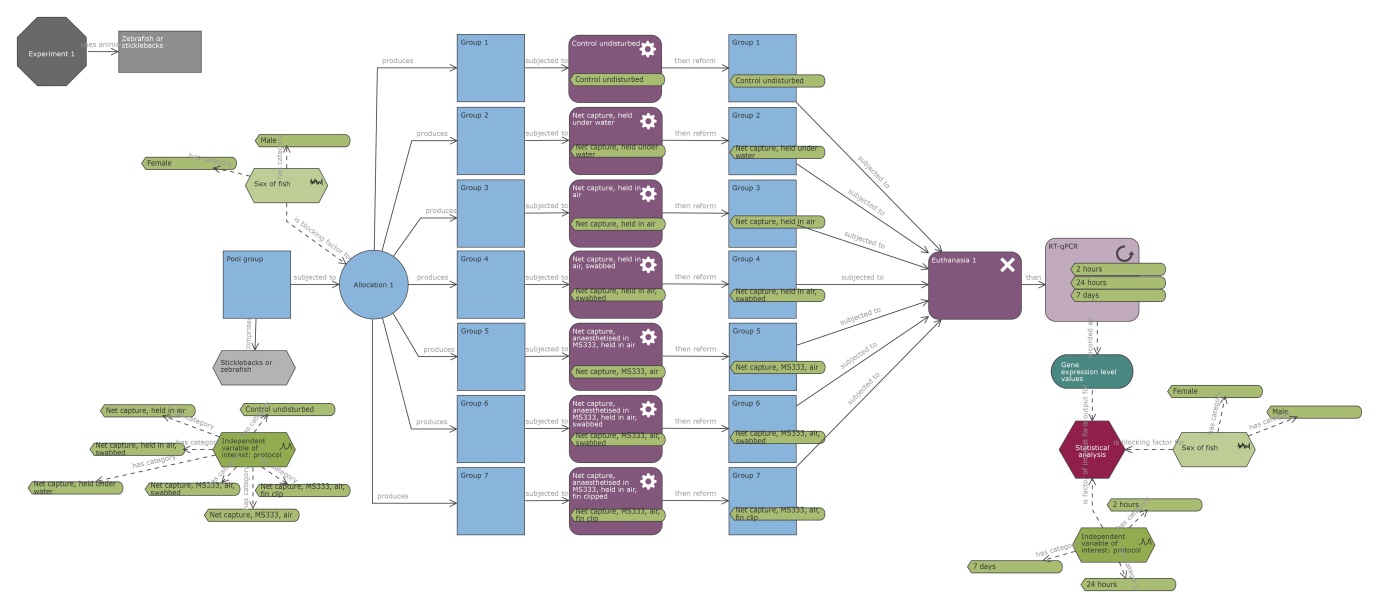


Supplemental tables

Tables showing statistical analysis of all data

Table S1 OBR and cortisol data. Significant values (p < 0.05) are shown in bold and highlighted.

| **Species** | **Indicator** | **Manipulation** | **df** | ***H*** | ***P*** |
| --- | --- | --- | --- | --- | --- |
| Stickleback | OBR | Netted underwater | 6 | 144.1 | 0.1054 |
|  |  | Netted air | 6 | 144.1 | 0.9091 |
|  |  | Swabbed | 6 | 144.1 | 0.2873 |
|  |  | MS222 | 6 | 144.1 | **0.0004** |
|  |  | MS222 + swabbed | 6 | 144.1 | **<0.0001** |
|  |  | MS222 + fin-clipped | 6 | 144.1 | **<0.0001** |
|  | Cortisol | Netted underwater | 6 | 16.77 | >0.9999 |
|  |  | Netted air | 6 | 16.77 | >0.9999 |
|  |  | Swabbed | 6 | 16.77 | >0.9999 |
|  |  | MS222 | 6 | 16.77 | >0.9999 |
|  |  | MS222 + swabbed | 6 | 16.77 | 0.4887 |
|  |  | MS222 + fin-clipped | 6 | 16.77 | **0.0082** |
| Zebrafish | OBR | Netted underwater | 6 | 65.45 | >0.9999 |
|  |  | Netted air | 6 | 65.45 | **0.0005** |
|  |  | Swabbed | 6 | 65.45 | 0.0582 |
|  |  | MS222 | 6 | 65.45 | >0.9999 |
|  |  | MS222 + swabbed | 6 | 65.45 | **<0.0001** |
|  |  | MS222 + fin-clipped | 6 | 65.45 | **<0.0001** |
|  | Cortisol | Netted underwater | 6 | 14.64 | 0.4621 |
|  |  | Netted air | 6 | 14.64 | 0.3574 |
|  |  | Swabbed | 6 | 14.64 | 0.7460 |
|  |  | MS222 | 6 | 14.64 | 0.4247 |
|  |  | MS222 + swabbed | 6 | 14.64 | >0.9999 |
|  |  | MS222 + fin-clipped | 6 | 14.64 | **0.0024** |

Table S2 Stickleback performance in the novel tank test, distance swum and time spent at the bottom. Significant values (p < 0.05) are shown in bold and higlighted.

| **Species** | **Indicator** | **Day** | **Manipulation** | **df** | ***H*** | ***P*** |
| --- | --- | --- | --- | --- | --- | --- |
| Stickleback | NTT Distance | 1 | Netted underwater | 6 | 7.229 | 0.3463 |
|  |  | 1 | Netted air | 6 | 7.229 | 0.4621 |
|  |  | 1 | Swabbed | 6 | 7.229 | >0.9999 |
|  |  | 1 | MS222 | 6 | 7.229 | 0.5106 |
|  |  | 1 | MS222 + swabbed | 6 | 7.229 | >0.9999 |
|  |  | 1 | MS222 + fin-clipped | 6 | 7.229 | >0.9999 |
|  |  | 2 | Netted underwater | 6 | 16.4 | **0.0078** |
|  |  | 2 | Netted air | 6 | 16.4 | **0.0036** |
|  |  | 2 | Swabbed | 6 | 16.4 | 0.0542 |
|  |  | 2 | MS222 | 6 | 16.4 | 0.2091 |
|  |  | 2 | MS222 + swabbed | 6 | 16.4 | **0.0106** |
|  |  | 2 | MS222 + fin-clipped | 6 | 16.4 | **0.0400** |
|  |  | 7 | Netted underwater | 6 | 5.547 | >0.9999 |
|  |  | 7 | Netted air | 6 | 5.547 | >0.9999 |
|  |  | 7 | Swabbed | 6 | 5.547 | >0.9999 |
|  |  | 7 | MS222 | 6 | 5.547 | 0.9175 |
|  |  | 7 | MS222 + swabbed | 6 | 5.547 | >0.9999 |
|  |  | 7 | MS222 + fin-clipped | 6 | 5.547 | >0.9999 |
|  | NTT Bottom | 1 | Netted underwater | 6 | 8.577 | >0.9999 |
|  |  | 1 | Netted air | 6 | 8.577 | 0.4792 |
|  |  | 1 | Swabbed | 6 | 8.577 | >0.9999 |
|  |  | 1 | MS222 | 6 | 8.577 | 0.4378 |
|  |  | 1 | MS222 + swabbed | 6 | 8.577 | 0.1701 |
|  |  | 1 | MS222 + fin-clipped | 6 | 8.577 | 0.9691 |
|  |  | 2 | Netted underwater | 6 | 3.993 | >0.9999 |
|  |  | 2 | Netted air | 6 | 3.993 | >0.9999 |
|  |  | 2 | Swabbed | 6 | 3.993 | >0.9999 |
|  |  | 2 | MS222 | 6 | 3.993 | >0.9999 |
|  |  | 2 | MS222 + swabbed | 6 | 3.993 | >0.9999 |
|  |  | 2 | MS222 + fin-clipped | 6 | 3.993 | 0.6449 |
|  |  | 7 | Netted underwater | 6 | 5.166 | >0.9999 |
|  |  | 7 | Netted air | 6 | 5.166 | 0.7366 |
|  |  | 7 | Swabbed | 6 | 5.166 | 0.5513 |
|  |  | 7 | MS222 | 6 | 5.166 | 0.5513 |
|  |  | 7 | MS222 + swabbed | 6 | 5.166 | >0.9999 |
|  |  | 7 | MS222 + fin-clipped | 6 | 5.166 | >0.9999 |

Table S3 Stickleback performance in the open field and light/dark tests, distance swum, time spent in the centre and time spent in the white zone. Significant values (p < 0.05) are shown in bold and highlighted.

| **Species** | **Indicator** | **Day** | **Manipulation** | **df** | ***H*** | ***P*** |
| --- | --- | --- | --- | --- | --- | --- |
| Stickleback | OF Distance | 1 | Netted underwater | 6 | 7.188 | >0.9999 |
|  |  | 1 | Netted air | 6 | 7.188 | >0.9999 |
|  |  | 1 | Swabbed | 6 | 7.188 | >0.9999 |
|  |  | 1 | MS222 | 6 | 7.188 | >0.9999 |
|  |  | 1 | MS222 + swabbed | 6 | 7.188 | >0.9999 |
|  |  | 1 | MS222 + fin-clipped | 6 | 7.188 | >0.9999 |
|  |  | 2 | Netted underwater | 6 | 6.422 | 0.8900 |
|  |  | 2 | Netted air | 6 | 6.422 | 0.1381 |
|  |  | 2 | Swabbed | 6 | 6.422 | >0.9999 |
|  |  | 2 | MS222 | 6 | 6.422 | 0.5598 |
|  |  | 2 | MS222 + swabbed | 6 | 6.422 | >0.9999 |
|  |  | 2 | MS222 + fin-clipped | 6 | 6.422 | >0.9999 |
|  |  | 7 | Netted underwater | 6 | 7.254 | >0.9999 |
|  |  | 7 | Netted air | 6 | 7.254 | >0.9999 |
|  |  | 7 | Swabbed | 6 | 7.254 | 0.3971 |
|  |  | 7 | MS222 | 6 | 7.254 | >0.9999 |
|  |  | 7 | MS222 + swabbed | 6 | 7.254 | >0.9999 |
|  |  | 7 | MS222 + fin-clipped | 6 | 7.254 | 0.9064 |
|  | OF Centre | 1 | Netted underwater | 6 | 4.79 | >0.9999 |
|  |  | 1 | Netted air | 6 | 4.79 | >0.9999 |
|  |  | 1 | Swabbed | 6 | 4.79 | >0.9999 |
|  |  | 1 | MS222 | 6 | 4.79 | >0.9999 |
|  |  | 1 | MS222 + swabbed | 6 | 4.79 | >0.9999 |
|  |  | 1 | MS222 + fin-clipped | 6 | 4.79 | >0.9999 |
|  |  | 2 | Netted underwater | 6 | 2.798 | >0.9999 |
|  |  | 2 | Netted air | 6 | 2.798 | >0.9999 |
|  |  | 2 | Swabbed | 6 | 2.798 | >0.9999 |
|  |  | 2 | MS222 | 6 | 2.798 | >0.9999 |
|  |  | 2 | MS222 + swabbed | 6 | 2.798 | >0.9999 |
|  |  | 2 | MS222 + fin-clipped | 6 | 2.798 | >0.9999 |
|  |  | 7 | Netted underwater | 6 | 3.468 | 0.7264 |
|  |  | 7 | Netted air | 6 | 3.468 | >0.9999 |
|  |  | 7 | Swabbed | 6 | 3.468 | >0.9999 |
|  |  | 7 | MS222 | 6 | 3.468 | >0.9999 |
|  |  | 7 | MS222 + swabbed | 6 | 3.468 | 0.7618 |
|  |  | 7 | MS222 + fin-clipped | 6 | 3.468 | >0.9999 |
|  | L/D White | 1 | Netted underwater | 6 | 15.57 | 0.2459 |
|  |  | 1 | Netted air | 6 | 15.57 | >0.9999 |
|  |  | 1 | Swabbed | 6 | 15.57 | >0.9999 |
|  |  | 1 | MS222 | 6 | 15.57 | **0.0498** |
|  |  | 1 | MS222 + swabbed | 6 | 15.57 | >0.9999 |
|  |  | 1 | MS222 + fin-clipped | 6 | 15.57 | >0.9999 |
|  |  | 2 | Netted underwater | 6 | 10.42 | >0.9999 |
|  |  | 2 | Netted air | 6 | 10.42 | >0.9999 |
|  |  | 2 | Swabbed | 6 | 10.42 | >0.9999 |
|  |  | 2 | MS222 | 6 | 10.42 | 0.3637 |
|  |  | 2 | MS222 + swabbed | 6 | 10.42 | 0.6021 |
|  |  | 2 | MS222 + fin-clipped | 6 | 10.42 | >0.9999 |
|  |  | 7 | Netted underwater | 6 | 8.63 | 0.7147 |
|  |  | 7 | Netted air | 6 | 8.63 | >0.9999 |
|  |  | 7 | Swabbed | 6 | 8.63 | >0.9999 |
|  |  | 7 | MS222 | 6 | 8.63 | >0.9999 |
|  |  | 7 | MS222 + swabbed | 6 | 8.63 | >0.9999 |
|  |  | 7 | MS222 + fin-clipped | 6 | 8.63 | >0.9999 |

Table S4 Zebrafish performance in the novel tank test, distance swum and time spent at the bottom. Significant values (p < 0.05) are shown in bold and highlighted.

| **Species** | **Indicator** | **Day** | **Manipulation** | **df** | ***H*** | ***P*** |
| --- | --- | --- | --- | --- | --- | --- |
| Zebrafish | NTT Distance | 1 | Netted underwater | 6 | 19.8 | >0.9999 |
|  |  | 1 | Netted air | 6 | 19.8 | >0.9999 |
|  |  | 1 | Swabbed | 6 | 19.8 | >0.9999 |
|  |  | 1 | MS222 | 6 | 19.8 | >0.9999 |
|  |  | 1 | MS222 + swabbed | 6 | 19.8 | 0.8873 |
|  |  | 1 | MS222 + fin-clipped | 6 | 19.8 | **0.0103** |
|  |  | 2 | Netted underwater | 6 | 19.53 | >0.9999 |
|  |  | 2 | Netted air | 6 | 19.53 | 0.7713 |
|  |  | 2 | Swabbed | 6 | 19.53 | **0.0055** |
|  |  | 2 | MS222 | 6 | 19.53 | >0.9999 |
|  |  | 2 | MS222 + swabbed | 6 | 19.53 | 0.3947 |
|  |  | 2 | MS222 + fin-clipped | 6 | 19.53 | 0.4664 |
|  |  | 7 | Netted underwater | 6 | 26.96 | **0.0211** |
|  |  | 7 | Netted air | 6 | 26.96 | <0.0001 |
|  |  | 7 | Swabbed | 6 | 26.96 | **0.0007** |
|  |  | 7 | MS222 | 6 | 26.96 | 0.7556 |
|  |  | 7 | MS222 + swabbed | 6 | 26.96 | **0.0117** |
|  |  | 7 | MS222 + fin-clipped | 6 | 26.96 | 0.0516 |
|  | NTT Bottom | 1 | Netted underwater | 6 | 34.83 | **0.0390** |
|  |  | 1 | Netted air | 6 | 34.83 | 0.1144 |
|  |  | 1 | Swabbed | 6 | 34.83 | >0.9999 |
|  |  | 1 | MS222 | 6 | 34.83 | 0.3488 |
|  |  | 1 | MS222 + swabbed | 6 | 34.83 | 0.1225 |
|  |  | 1 | MS222 + fin-clipped | 6 | 34.83 | >0.9999 |
|  |  | 2 | Netted underwater | 6 | 45.1 | 0.1290 |
|  |  | 2 | Netted air | 6 | 45.1 | **0.0009** |
|  |  | 2 | Swabbed | 6 | 45.1 | **<0.0001** |
|  |  | 2 | MS222 | 6 | 45.1 | >0.9999 |
|  |  | 2 | MS222 + swabbed | 6 | 45.1 | **0.0190** |
|  |  | 2 | MS222 + fin-clipped | 6 | 45.1 | >0.9999 |
|  |  | 7 | Netted underwater | 6 | 39.79 | **0.0204** |
|  |  | 7 | Netted air | 6 | 39.79 | **<0.0001** |
|  |  | 7 | Swabbed | 6 | 39.79 | **0.0005** |
|  |  | 7 | MS222 | 6 | 39.79 | 0.8680 |
|  |  | 7 | MS222 + swabbed | 6 | 39.79 | **<0.0001** |
|  |  | 7 | MS222 + fin-clipped | 6 | 39.79 | >0.9999 |

Table S5 Zebrafish performance in the open field (OF) and light/dark (L/D) tests, distance swum, time spent in the centre and time spent in the white zone. Significant values (p < 0.05) are shown in bold and highlighted.

| **Species** | **Indicator** | **Day** | **Manipulation** | **df** | ***H*** | ***P*** |
| --- | --- | --- | --- | --- | --- | --- |
| Zebrafish | OF Distance | 1 | Netted underwater | 6 | 25.25 | 0.2678 |
|  |  | 1 | Netted air | 6 | 25.25 | 0.5751 |
|  |  | 1 | Swabbed | 6 | 25.25 | 0.4535 |
|  |  | 1 | MS222 | 6 | 25.25 | >0.9999 |
|  |  | 1 | MS222 + swabbed | 6 | 25.25 | 0.1496 |
|  |  | 1 | MS222 + fin-clipped | 6 | 25.25 | **0.0015** |
|  |  | 2 | Netted underwater | 6 | 10.36 | >0.9999 |
|  |  | 2 | Netted air | 6 | 10.36 | >0.9999 |
|  |  | 2 | Swabbed | 6 | 10.36 | 0.3818 |
|  |  | 2 | MS222 | 6 | 10.36 | >0.9999 |
|  |  | 2 | MS222 + swabbed | 6 | 10.36 | 0.5475 |
|  |  | 2 | MS222 + fin-clipped | 6 | 10.36 | 0.3165 |
|  |  | 7 | Netted underwater | 6 | 6.866 | >0.9999 |
|  |  | 7 | Netted air | 6 | 6.866 | >0.9999 |
|  |  | 7 | Swabbed | 6 | 6.866 | >0.9999 |
|  |  | 7 | MS222 | 6 | 6.866 | >0.9999 |
|  |  | 7 | MS222 + swabbed | 6 | 6.866 | >0.9999 |
|  |  | 7 | MS222 + fin-clipped | 6 | 6.866 | >0.9999 |
|  | OF Centre | 1 | Netted underwater | 6 | 10.35 | **0.0378** |
|  |  | 1 | Netted air | 6 | 10.35 | 0.1704 |
|  |  | 1 | Swabbed | 6 | 10.35 | >0.9999 |
|  |  | 1 | MS222 | 6 | 10.35 | 0.5060 |
|  |  | 1 | MS222 + swabbed | 6 | 10.35 | >0.9999 |
|  |  | 1 | MS222 + fin-clipped | 6 | 10.35 | 0.1725 |
|  |  | 2 | Netted underwater | 6 | 15.33 | >0.9999 |
|  |  | 2 | Netted air | 6 | 15.33 | **0.0206** |
|  |  | 2 | Swabbed | 6 | 15.33 | >0.9999 |
|  |  | 2 | MS222 | 6 | 15.33 | 0.2645 |
|  |  | 2 | MS222 + swabbed | 6 | 15.33 | 0.3100 |
|  |  | 2 | MS222 + fin-clipped | 6 | 15.33 | 0.9554 |
|  |  | 7 | Netted underwater | 6 | 11.32 | >0.9999 |
|  |  | 7 | Netted air | 6 | 11.32 | 0.1590 |
|  |  | 7 | Swabbed | 6 | 11.32 | >0.9999 |
|  |  | 7 | MS222 | 6 | 11.32 | **0.0310** |
|  |  | 7 | MS222 + swabbed | 6 | 11.32 | 0.1866 |
|  |  | 7 | MS222 + fin-clipped | 6 | 11.32 | 0.7556 |
|  | L/D White | 1 | Netted underwater | 6 | 31.39 | **0.0089** |
|  |  | 1 | Netted air | 6 | 31.39 | 0.5946 |
|  |  | 1 | Swabbed | 6 | 31.39 | >0.9999 |
|  |  | 1 | MS222 | 6 | 31.39 | >0.9999 |
|  |  | 1 | MS222 + swabbed | 6 | 31.39 | **0.0307** |
|  |  | 1 | MS222 + fin-clipped | 6 | 31.39 | 0.1805 |
|  |  | 2 | Netted underwater | 6 | 33.94 | **0.0143** |
|  |  | 2 | Netted air | 6 | 33.94 | 0.0678 |
|  |  | 2 | Swabbed | 6 | 33.94 | 0.0685 |
|  |  | 2 | MS222 | 6 | 33.94 | 0.7958 |
|  |  | 2 | MS222 + swabbed | 6 | 33.94 | **0.0277** |
|  |  | 2 | MS222 + fin-clipped | 6 | 33.94 | >0.9999 |
|  |  | 7 | Netted underwater | 6 | 13.08 | 0.7274 |
|  |  | 7 | Netted air | 6 | 13.08 | 0.8053 |
|  |  | 7 | Swabbed | 6 | 13.08 | 0.3656 |
|  |  | 7 | MS222 | 6 | 13.08 | >0.9999 |
|  |  | 7 | MS222 + swabbed | 6 | 13.08 | **0.0128** |
|  |  | 7 | MS222 + fin-clipped | 6 | 13.08 | 0.3498 |

Table S6 Stickleback expression level of the marker genes; *brain-derived neurotrophic factor* (*bdnf*), *corticotropin releasing hormone a* (*crha*), *corticotropin releasing hormone b* (*crhb*), *galanin* (*galn*) and *neuropeptide y* (*npy*)(. Significant values (p < 0.05) are shown in bold and highlighted.

| **Species** | **Gene** | **Day** | **Manipulation** | **df** | ***H*** | ***P*** |
| --- | --- | --- | --- | --- | --- | --- |
| Stickleback | *bdnf* | 1 | Netted underwater | 6 | 10.89 | >0.9999 |
|  |  | 1 | Netted air | 6 | 10.89 | >0.9999 |
|  |  | 1 | Swabbed | 6 | 10.89 | 0.7113 |
|  |  | 1 | MS222 | 6 | 10.89 | >0.9999 |
|  |  | 1 | MS222 + swabbed | 6 | 10.89 | >0.9999 |
|  |  | 1 | MS222 + fin-clipped | 6 | 10.89 | **0.0349** |
|  |  | 2 | Netted underwater | 6 | 8.639 | >0.9999 |
|  |  | 2 | Netted air | 6 | 8.639 | 0.6055 |
|  |  | 2 | Swabbed | 6 | 8.639 | 0.6879 |
|  |  | 2 | MS222 | 6 | 8.639 | >0.9999 |
|  |  | 2 | MS222 + swabbed | 6 | 8.639 | >0.9999 |
|  |  | 2 | MS222 + fin-clipped | 6 | 8.639 | 0.1869 |
|  |  | 7 | Netted underwater | 6 | 5.45 | >0.9999 |
|  |  | 7 | Netted air | 6 | 5.45 | 0.4731 |
|  |  | 7 | Swabbed | 6 | 5.45 | >0.9999 |
|  |  | 7 | MS222 | 6 | 5.45 | >0.9999 |
|  |  | 7 | MS222 + swabbed | 6 | 5.45 | >0.9999 |
|  |  | 7 | MS222 + fin-clipped | 6 | 5.45 | 0.2822 |
|  | *crha* | 1 | Netted underwater | 6 | 37.78 | >0.9999 |
|  |  | 1 | Netted air | 6 | 37.78 | **0.0013** |
|  |  | 1 | Swabbed | 6 | 37.78 | **0.0006** |
|  |  | 1 | MS222 | 6 | 37.78 | 0.1001 |
|  |  | 1 | MS222 + swabbed | 6 | 37.78 | **0.0181** |
|  |  | 1 | MS222 + fin-clipped | 6 | 37.78 | **<0.0001** |
|  |  | 2 | Netted underwater | 6 | 30.88 | >0.9999 |
|  |  | 2 | Netted air | 6 | 30.88 | **0.0002** |
|  |  | 2 | Swabbed | 6 | 30.88 | **0.0417** |
|  |  | 2 | MS222 | 6 | 30.88 | 0.1495 |
|  |  | 2 | MS222 + swabbed | 6 | 30.88 | 0.2545 |
|  |  | 2 | MS222 + fin-clipped | 6 | 30.88 | **<0.0001** |
|  |  | 7 | Netted underwater | 6 | 26.3 | >0.9999 |
|  |  | 7 | Netted air | 6 | 26.3 | **0.0009** |
|  |  | 7 | Swabbed | 6 | 26.3 | **0.0028** |
|  |  | 7 | MS222 | 6 | 26.3 | 0.0586 |
|  |  | 7 | MS222 + swabbed | 6 | 26.3 | 0.1205 |
|  |  | 7 | MS222 + fin-clipped | 6 | 26.3 | **0.0002** |
|  | *crhb* | 1 | Netted underwater | 6 | 38.72 | >0.9999 |
|  |  | 1 | Netted air | 6 | 38.72 | **0.0014** |
|  |  | 1 | Swabbed | 6 | 38.72 | **0.0011** |
|  |  | 1 | MS222 | 6 | 38.72 | **0.0412** |
|  |  | 1 | MS222 + swabbed | 6 | 38.72 | **0.0086** |
|  |  | 1 | MS222 + fin-clipped | 6 | 38.72 | **0.0001** |
|  |  | 2 | Netted underwater | 6 | 34.63 | >0.9999 |
|  |  | 2 | Netted air | 6 | 34.63 | **0.0002** |
|  |  | 2 | Swabbed | 6 | 34.63 | **0.0020** |
|  |  | 2 | MS222 | 6 | 34.63 | **0.0169** |
|  |  | 2 | MS222 + swabbed | 6 | 34.63 | 0.1147 |
|  |  | 2 | MS222 + fin-clipped | 6 | 34.63 | **0.0010** |
|  |  | 7 | Netted underwater | 6 | 39.34 | >0.9999 |
|  |  | 7 | Netted air | 6 | 39.34 | **<0.0001** |
|  |  | 7 | Swabbed | 6 | 39.34 | **0.0002** |
|  |  | 7 | MS222 | 6 | 39.34 | **0.0088** |
|  |  | 7 | MS222 + swabbed | 6 | 39.34 | **0.0047** |
|  |  | 7 | MS222 + fin-clipped | 6 | 39.34 | **0.0002** |
|  | *galn* | 1 | Netted underwater | 6 | 15.18 | >0.9999 |
|  |  | 1 | Netted air | 6 | 15.18 | 0.2163 |
|  |  | 1 | Swabbed | 6 | 15.18 | 0.3101 |
|  |  | 1 | MS222 | 6 | 15.18 | >0.9999 |
|  |  | 1 | MS222 + swabbed | 6 | 15.18 | 0.5601 |
|  |  | 1 | MS222 + fin-clipped | 6 | 15.18 | **0.0043** |
|  |  | 2 | Netted underwater | 6 | 13.55 | >0.9999 |
|  |  | 2 | Netted air | 6 | 13.55 | 0.1793 |
|  |  | 2 | Swabbed | 6 | 13.55 | >0.9999 |
|  |  | 2 | MS222 | 6 | 13.55 | >0.9999 |
|  |  | 2 | MS222 + swabbed | 6 | 13.55 | >0.9999 |
|  |  | 2 | MS222 + fin-clipped | 6 | 13.55 | 0.6215 |
|  |  | 7 | Netted underwater | 6 | 7.416 | >0.9999 |
|  |  | 7 | Netted air | 6 | 7.416 | >0.9999 |
|  |  | 7 | Swabbed | 6 | 7.416 | 0.9738 |
|  |  | 7 | MS222 | 6 | 7.416 | >0.9999 |
|  |  | 7 | MS222 + swabbed | 6 | 7.416 | >0.9999 |
|  |  | 7 | MS222 + fin-clipped | 6 | 7.416 | >0.9999 |
|  | *npy* | 1 | Netted underwater | 6 | 43.83 | >0.9999 |
|  |  | 1 | Netted air | 6 | 43.83 | **<0.0001** |
|  |  | 1 | Swabbed | 6 | 43.83 | **0.0001** |
|  |  | 1 | MS222 | 6 | 43.83 | **0.0079** |
|  |  | 1 | MS222 + swabbed | 6 | 43.83 | **0.0022** |
|  |  | 1 | MS222 + fin-clipped | 6 | 43.83 | **<0.0001** |
|  |  | 2 | Netted underwater | 6 | 34.94 | >0.9999 |
|  |  | 2 | Netted air | 6 | 34.94 | **0.0009** |
|  |  | 2 | Swabbed | 6 | 34.94 | **0.0016** |
|  |  | 2 | MS222 | 6 | 34.94 | **0.0341** |
|  |  | 2 | MS222 + swabbed | 6 | 34.94 | 0.2012 |
|  |  | 2 | MS222 + fin-clipped | 6 | 34.94 | **0.0024** |
|  |  | 7 | Netted underwater | 6 | 31.27 | >0.9999 |
|  |  | 7 | Netted air | 6 | 31.27 | **0.0003** |
|  |  | 7 | Swabbed | 6 | 31.27 | **0.0001** |
|  |  | 7 | MS222 | 6 | 31.27 | **0.0069** |
|  |  | 7 | MS222 + swabbed | 6 | 31.27 | **0.0192** |
|  |  | 7 | MS222 + fin-clipped | 6 | 31.27 | **0.0019** |

Table S7 Zebrafish expression level of the marker genes; *brain-derived neurotrophic factor* (*bdnf*), *corticotropin releasing hormone a* (*crha*), *corticotropin releasing hormone b* (*crhb*), *galanin* (*galn*) and *neuropeptide y* (*npy*)(. Significant values (p < 0.05) are shown in bold and highlighted.

| **Species** | **Gene** | **Day** | **Manipulation** | **df** | ***H*** | ***P*** |
| --- | --- | --- | --- | --- | --- | --- |
| Zebrafish | *bdnf* | 1 | Netted underwater | 6 | 16.31 | >0.9999 |
|  |  | 1 | Netted air | 6 | 16.31 | >0.9999 |
|  |  | 1 | Swabbed | 6 | 16.31 | >0.9999 |
|  |  | 1 | MS222 | 6 | 16.31 | 0.1310 |
|  |  | 1 | MS222 + swabbed | 6 | 16.31 | 0.1882 |
|  |  | 1 | MS222 + fin-clipped | 6 | 16.31 | **0.0151** |
|  |  | 2 | Netted underwater | 6 | 15.28 | 0.2537 |
|  |  | 2 | Netted air | 6 | 15.28 | >0.9999 |
|  |  | 2 | Swabbed | 6 | 15.28 | **0.0206** |
|  |  | 2 | MS222 | 6 | 15.28 | 0.4276 |
|  |  | 2 | MS222 + swabbed | 6 | 15.28 | **0.0331** |
|  |  | 2 | MS222 + fin-clipped | 6 | 15.28 | 0.8094 |
|  |  | 7 | Netted underwater | 6 | 7.272 | >0.9999 |
|  |  | 7 | Netted air | 6 | 7.272 | >0.9999 |
|  |  | 7 | Swabbed | 6 | 7.272 | 0.2140 |
|  |  | 7 | MS222 | 6 | 7.272 | >0.9999 |
|  |  | 7 | MS222 + swabbed | 6 | 7.272 | >0.9999 |
|  |  | 7 | MS222 + fin-clipped | 6 | 7.272 | >0.9999 |
|  | *crha* | 1 | Netted underwater | 6 | 29.03 | >0.9999 |
|  |  | 1 | Netted air | 6 | 29.03 | **0.0097** |
|  |  | 1 | Swabbed | 6 | 29.03 | >0.9999 |
|  |  | 1 | MS222 | 6 | 29.03 | >0.9999 |
|  |  | 1 | MS222 + swabbed | 6 | 29.03 | >0.9999 |
|  |  | 1 | MS222 + fin-clipped | 6 | 29.03 | **0.0032** |
|  |  | 2 | Netted underwater | 6 | 13.22 | 0.0622 |
|  |  | 2 | Netted air | 6 | 13.22 | 0.0550 |
|  |  | 2 | Swabbed | 6 | 13.22 | >0.9999 |
|  |  | 2 | MS222 | 6 | 13.22 | >0.9999 |
|  |  | 2 | MS222 + swabbed | 6 | 13.22 | >0.9999 |
|  |  | 2 | MS222 + fin-clipped | 6 | 13.22 | >0.9999 |
|  |  | 7 | Netted underwater | 6 | 22.36 | 0.1116 |
|  |  | 7 | Netted air | 6 | 22.36 | 0.6620 |
|  |  | 7 | Swabbed | 6 | 22.36 | 0.6785 |
|  |  | 7 | MS222 | 6 | 22.36 | >0.9999 |
|  |  | 7 | MS222 + swabbed | 6 | 22.36 | >0.9999 |
|  |  | 7 | MS222 + fin-clipped | 6 | 22.36 | >0.9999 |
|  | *crhb* | 1 | Netted underwater | 6 | 23.54 | 0.5453 |
|  |  | 1 | Netted air | 6 | 23.54 | >0.9999 |
|  |  | 1 | Swabbed | 6 | 23.54 | **0.0321** |
|  |  | 1 | MS222 | 6 | 23.54 | >0.9999 |
|  |  | 1 | MS222 + swabbed | 6 | 23.54 | >0.9999 |
|  |  | 1 | MS222 + fin-clipped | 6 | 23.54 | >0.9999 |
|  |  | 2 | Netted underwater | 6 | 23.92 | 0.0811 |
|  |  | 2 | Netted air | 6 | 23.92 | >0.9999 |
|  |  | 2 | Swabbed | 6 | 23.92 | >0.9999 |
|  |  | 2 | MS222 | 6 | 23.92 | >0.9999 |
|  |  | 2 | MS222 + swabbed | 6 | 23.92 | >0.9999 |
|  |  | 2 | MS222 + fin-clipped | 6 | 23.92 | 0.2766 |
|  |  | 7 | Netted underwater | 6 | 20.33 | 0.8693 |
|  |  | 7 | Netted air | 6 | 20.33 | 0.6902 |
|  |  | 7 | Swabbed | 6 | 20.33 | >0.9999 |
|  |  | 7 | MS222 | 6 | 20.33 | 0.3096 |
|  |  | 7 | MS222 + swabbed | 6 | 20.33 | 0.0809 |
|  |  | 7 | MS222 + fin-clipped | 6 | 20.33 | >0.9999 |
|  | *galn* | 1 | Netted underwater | 6 | 11.81 | >0.9999 |
|  |  | 1 | Netted air | 6 | 11.81 | >0.9999 |
|  |  | 1 | Swabbed | 6 | 11.81 | 0.2975 |
|  |  | 1 | MS222 | 6 | 11.81 | >0.9999 |
|  |  | 1 | MS222 + swabbed | 6 | 11.81 | >0.9999 |
|  |  | 1 | MS222 + fin-clipped | 6 | 11.81 | >0.9999 |
|  |  | 2 | Netted underwater | 6 | 6.913 | >0.9999 |
|  |  | 2 | Netted air | 6 | 6.913 | >0.9999 |
|  |  | 2 | Swabbed | 6 | 6.913 | >0.9999 |
|  |  | 2 | MS222 | 6 | 6.913 | >0.9999 |
|  |  | 2 | MS222 + swabbed | 6 | 6.913 | >0.9999 |
|  |  | 2 | MS222 + fin-clipped | 6 | 6.913 | >0.9999 |
|  |  | 7 | Netted underwater | 6 | 3.788 | >0.9999 |
|  |  | 7 | Netted air | 6 | 3.788 | >0.9999 |
|  |  | 7 | Swabbed | 6 | 3.788 | >0.9999 |
|  |  | 7 | MS222 | 6 | 3.788 | >0.9999 |
|  |  | 7 | MS222 + swabbed | 6 | 3.788 | >0.9999 |
|  |  | 7 | MS222 + fin-clipped | 6 | 3.788 | >0.9999 |
|  | *npy* | 1 | Netted underwater | 6 | 25.52 | 0.1939 |
|  |  | 1 | Netted air | 6 | 25.52 | 0.1249 |
|  |  | 1 | Swabbed | 6 | 25.52 | >0.9999 |
|  |  | 1 | MS222 | 6 | 25.52 | 0.0690 |
|  |  | 1 | MS222 + swabbed | 6 | 25.52 | 0.1592 |
|  |  | 1 | MS222 + fin-clipped | 6 | 25.52 | **0.0483** |
|  |  | 2 | Netted underwater | 6 | 12.23 | 0.0690 |
|  |  | 2 | Netted air | 6 | 12.23 | >0.9999 |
|  |  | 2 | Swabbed | 6 | 12.23 | >0.9999 |
|  |  | 2 | MS222 | 6 | 12.23 | 0.9137 |
|  |  | 2 | MS222 + swabbed | 6 | 12.23 | 0.9832 |
|  |  | 2 | MS222 + fin-clipped | 6 | 12.23 | >0.9999 |
|  |  | 7 | Netted underwater | 6 | 20.7 | 0.0642 |
|  |  | 7 | Netted air | 6 | 20.7 | 0.4896 |
|  |  | 7 | Swabbed | 6 | 20.7 | >0.9999 |
|  |  | 7 | MS222 | 6 | 20.7 | >0.9999 |
|  |  | 7 | MS222 + swabbed | 6 | 20.7 | >0.9999 |
|  |  | 7 | MS222 + fin-clipped | 6 | 20.7 | >0.9999 |

Table S8 Health and condition indicators; Length, weight, hepatosomatic (HSI), splenosomatic (SSI), gonadosomatic (GSI) and nephrosomatic (NSI) indices. Significant values (p < 0.05) are shown in bold and highlighted.

| **Species** | **Indicator** | **Manipulation** | **df** | ***H*** | ***P*** |
| --- | --- | --- | --- | --- | --- |
| Stickleback | Length | Netted underwater | 6 | 52.85 | 0.9943 |
|  |  | Netted air | 6 | 52.85 | **0.0394** |
|  |  | Swabbed | 6 | 52.85 | >0.9999 |
|  |  | MS222 | 6 | 52.85 | **0.0224** |
|  |  | MS222 + swabbed | 6 | 52.85 | **0.0001** |
|  |  | MS222 + fin-clipped | 6 | 52.85 | **<0.0001** |
|  | Weight | Netted underwater | 6 | 42.85 | >0.9999 |
|  |  | Netted air | 6 | 42.85 | **0.0405** |
|  |  | Swabbed | 6 | 42.85 | >0.9999 |
|  |  | MS222 | 6 | 42.85 | **0.0252** |
|  |  | MS222 + swabbed | 6 | 42.85 | **0.0021** |
|  |  | MS222 + fin-clipped | 6 | 42.85 | **<0.0001** |
|  | HSI | Netted underwater | 6 | 9.397 | 0.2396 |
|  |  | Netted air | 6 | 9.397 | >0.9999 |
|  |  | Swabbed | 6 | 9.397 | >0.9999 |
|  |  | MS222 | 6 | 9.397 | >0.9999 |
|  |  | MS222 + swabbed | 6 | 9.397 | >0.9999 |
|  |  | MS222 + fin-clipped | 6 | 9.397 | 0.2126 |
|  | SSI | Netted underwater | 6 | 5.282 | 0.3415 |
|  |  | Netted air | 6 | 5.282 | >0.9999 |
|  |  | Swabbed | 6 | 5.282 | 0.9683 |
|  |  | MS222 | 6 | 5.282 | >0.9999 |
|  |  | MS222 + swabbed | 6 | 5.282 | >0.9999 |
|  |  | MS222 + fin-clipped | 6 | 5.282 | >0.9999 |
|  | GSI | Netted underwater | 6 | 6.971 | >0.9999 |
|  |  | Netted air | 6 | 6.971 | >0.9999 |
|  |  | Swabbed | 6 | 6.971 | 0.3941 |
|  |  | MS222 | 6 | 6.971 | >0.9999 |
|  |  | MS222 + swabbed | 6 | 6.971 | >0.9999 |
|  |  | MS222 + fin-clipped | 6 | 6.971 | >0.9999 |
|  | NSI | Netted underwater | 6 | 3.807 | >0.9999 |
|  |  | Netted air | 6 | 3.807 | >0.9999 |
|  |  | Swabbed | 6 | 3.807 | >0.9999 |
|  |  | MS222 | 6 | 3.807 | >0.9999 |
|  |  | MS222 + swabbed | 6 | 3.807 | >0.9999 |
|  |  | MS222 + fin-clipped | 6 | 3.807 | >0.9999 |
| Zebrafish | Length | Netted underwater | 6 | 23.87 | >0.9999 |
|  |  | Netted air | 6 | 23.87 | >0.9999 |
|  |  | Swabbed | 6 | 23.87 | 0.0537 |
|  |  | MS222 | 6 | 23.87 | >0.9999 |
|  |  | MS222 + swabbed | 6 | 23.87 | 0.3131 |
|  |  | MS222 + fin-clipped | 6 | 23.87 | >0.9999 |
|  | Weight | Netted underwater | 6 | 20.53 | >0.9999 |
|  |  | Netted air | 6 | 20.53 | 0.1937 |
|  |  | Swabbed | 6 | 20.53 | 0.7436 |
|  |  | MS222 | 6 | 20.53 | 0.5471 |
|  |  | MS222 + swabbed | 6 | 20.53 | 0.3047 |
|  |  | MS222 + fin-clipped | 6 | 20.53 | >0.9999 |
|  | HSI | Netted underwater | 6 | 24.45 | >0.9999 |
|  |  | Netted air | 6 | 24.45 | **0.0149** |
|  |  | Swabbed | 6 | 24.45 | 0.1965 |
|  |  | MS222 | 6 | 24.45 | **0.0003** |
|  |  | MS222 + swabbed | 6 | 24.45 | 0.1138 |
|  |  | MS222 + fin-clipped | 6 | 24.45 | **0.0052** |
|  | SSI | Netted underwater | 6 | 13.5 | 0.7033 |
|  |  | Netted air | 6 | 13.5 | >0.9999 |
|  |  | Swabbed | 6 | 13.5 | >0.9999 |
|  |  | MS222 | 6 | 13.5 | >0.9999 |
|  |  | MS222 + swabbed | 6 | 13.5 | >0.9999 |
|  |  | MS222 + fin-clipped | 6 | 13.5 | >0.9999 |
|  | GSI | Netted underwater | 6 | 3.853 | >0.9999 |
|  |  | Netted air | 6 | 3.853 | >0.9999 |
|  |  | Swabbed | 6 | 3.853 | >0.9999 |
|  |  | MS222 | 6 | 3.853 | >0.9999 |
|  |  | MS222 + swabbed | 6 | 3.853 | >0.9999 |
|  |  | MS222 + fin-clipped | 6 | 3.853 | 0.8442 |
|  | NSI | Netted underwater | 6 | 3.497 | >0.9999 |
|  |  | Netted air | 6 | 3.497 | >0.9999 |
|  |  | Swabbed | 6 | 3.497 | >0.9999 |
|  |  | MS222 | 6 | 3.497 | >0.9999 |
|  |  | MS222 + swabbed | 6 | 3.497 | >0.9999 |
|  |  | MS222 + fin-clipped | 6 | 3.497 | >0.9999 |

Supplemental references

1 Bitetti, A. *et al.* MicroRNA degradation by a conserved target RNA regulates animal behavior. *Nature structural & molecular biology* **25**, 244-251 (2018).

2 Carreño, G. H. *et al.* Nitric oxide interacts with monoamine oxidase to modulate aggression and anxiety-like behaviour. *European neuropsychopharmacology: the journal of the European College of Neuropsychopharmacology* (2017).

3 Craig-Bennett, A. The reproductive cycle of the three-spined stickleback, *Gasterosteus aculeatus*, Linn. *Philosophical Transactions of the Royal Society of London. Series B, Containing Papers of a Biological Character* **219**, 197-279 (1931).

4 Perlberg, S., Diamant, A., Ofir, R. & Zilberg, D. Characterization of swim bladder non‐inflation (SBN) in angelfish, *Pterophyllum scalare* (Schultz), and the effect of exposure to methylene blue. *Journal of Fish Diseases* **31**, 215-228 (2008).

5 OECD. Test No. 234: Fish Sexual Development Test, OECD Guidelines for the Testing of Chemicals, Section 2. (2011).
